# Effects of Duodenal Infusion of L-Citrulline on Plasma Metabolism, Fecal Microbiota Structure, and Reproductive Hormones in Ewes

**DOI:** 10.3390/life16071055

**Published:** 2026-06-24

**Authors:** Tingting Lu, Hui Chen, Jiaqi Liu, Tingting Li, Hao Lu, Reylağül Rehim, Haibo Lv, Chenyang Gao, Guodong Zhao

**Affiliations:** 1 College of Animal Science, Xinjiang Agricultural University, Urumqi 830052, China; lutt11071024@163.com (T.L.);; 2 Huishang Ecological Animal Husbandry Co., Ltd., Toksun County, Turpan 838100, China

**Keywords:** L-Cit, amino acid content, fecal microbiota structure, plasma metabolites, reproductive hormones

## Abstract

This experiment aimed to investigate the metabolism of L-Citrulline (L-Cit) in the intestinal tract of ewes and its effects on fecal microbiota composition, plasma metabolism, and reproductive hormone levels. Twelve 18-month-old non-pregnant multiparous Turpan black ewes weighing 51.65 kg ± 2.49 kg were selected and randomly assigned to a control group (Con) and an experimental group (L-Cit), with six ewes in each group. Both groups were fed identical nutrient-dense rations. In the Con group, 100 mL of saline was administered through the duodenal fistula, while the L-Cit group received an additional 0.25 g/kg BW^−1^ of L-Cit solution. On day 7, the crude protein and amino acid concentrations in feces and urine were assessed using total feces and urine collection methods. Fecal and blood samples were collected to evaluate microbiological and reproductive hormone indices, with blood samples also collected for plasma non-targeted metabolomics analysis two hours post-infusion. Compared to the Con group, the L-Cit group exhibited a significant reduction in crude protein content in feces (*p* < 0.05) and a highly significant decrease in urine (*p* < 0.01). Nitrogen metabolism indices did not differ significantly between groups (*p* > 0.05), but the L-lysine content in feces was significantly higher in the L-Cit group (*p* < 0.05). 16S rRNA sequencing revealed no significant PCA separation between the two groups. However, the relative abundance of *Lachnospiraceae_NK3A20_group*, *Oscillibacter*, and *Mogibacterium* was significantly higher in the Con group (*p* < 0.01), while *SP3-e08*, *Parvibacter*, *Anaerosporobacter*, *Butyricimonas*, and *Peptococcus* were more abundant in the L-Cit group (*p* < 0.05). LC-MS analysis showed significant up-regulation of purine and nucleotide metabolism pathways in the L-Cit group (*p* < 0.05). Plasma levels of estradiol (E_2_), progesterone (P_4_), gonadotropin-releasing hormone (GnRH), follicle-stimulating hormone (FSH), and luteinizing hormone (LH) were significantly elevated in the L-Cit group at both 1 and 2 h post-infusion (*p* < 0.01). These results suggest that duodenal infusion of L-Cit enhances intestinal nitrogen utilization, alters specific bacterial populations, promotes purine and nucleotide metabolism, and stimulates reproductive hormone secretion in ewes.

## 1. Introduction

L-Citrulline (L-Cit) is an α-amino acid first isolated from watermelon. It is a non-essential amino acid that can be synthesized endogenously via the urea and nitric oxide cycles [1,2]. L-Cit is a key intermediate in the urea cycle, involved in converting ammonia to urea for excretion. Although it does not participate in protein synthesis, it plays an important role in protein modification [3]. Absorbed by the small intestine and transported to the kidneys for arginine synthesis, it regulates body and plasma arginine levels [1]. Furthermore, L-Cit is a byproduct of nitric oxide (NO) production from arginine catalyzed by nitric oxide synthase [4], relying on argininosuccinate synthetase (ASS) and lyase (ASL) in kidney and intestinal cells for continuous NO generation, crucial for cardiovascular function, immune regulation, and neural signaling [5]. L-Cit alleviates oxidative stress by scavenging reactive oxygen species (ROS), protecting cells from oxidative damage [6]; improves blood flow and energy metabolism by increasing arginine/NO levels [7]; and, via NO-mediated vasodilation, improves penile blood flow, aiding in the treatment of mild erectile dysfunction [8]. In ruminants, L-Cit is synthesized by the intestine, primarily for renal arginine synthesis to meet the body’s arginine requirements [9]. The synthesis of arginine from L-Cit requires specific enzymes in the small intestine, thus playing a vital role in the endogenous synthesis of arginine in mammals. Zhao [10] found that L-Cit supplementation regulated protein digestion and absorption pathways, improved serum amino acid metabolism, and enhanced semen quality in rams. Fan [11] reported that L-Cit supplementation significantly increased sperm density and intestinal microbiota abundance in rams. Ma [12] found that supplementing different doses of L-Cit increased plasma concentrations of certain amino acids and reproductive hormones as well as antioxidant capacity in ewes, with 10 g/d L-Cit improving lambing rate and twin rate, indicating L-Cit’s important role in regulating animal reproductive performance. Moreover, L-Cit is typically not degraded by rumen microbes in ruminants, allowing it to reach the intestine intact, where it is further utilized via host metabolic pathways or the action of intestinal microbiota [13,14]. While oral supplementation is the practical application route, duodenal infusion in this study was employed as a precise experimental model to deliver a controlled dose directly to the absorptive site, thereby completely bypassing any potential ruminal interference and allowing for an accurate assessment of the dose-specific metabolic and endocrine effects of L-Cit. Therefore, this study used ewes to investigate the effects of duodenal infusion of L-Cit on plasma metabolism, fecal microbiota structure, and reproductive hormones in ewes using permanent proximal duodenal cannulation, providing a theoretical basis for the application of L-Cit in ruminant production.

## 2. Materials and Methods

### 2.1. Ethic Statement

All animal care and handling procedures in this study were conducted under the guidance of the *Guide for the Care and Use of Laboratory Animals*, China, and were approved by (protocol number: 2020022) the Animal Care Committee of Xinjiang Agricultural University (Urumqi, Xinjiang, China).

### 2.2. Experimental Materials

L-Citrulline (L-Cit) was purchased from Shandong Pingju Biotechnology Co., Ltd. (Jining, China), with a purity of 99.1%, ash content of 0.05%, and nitrogen content of 24%. A centrifuge (Model 5424) was obtained from Eppendorf AG (Hamburg, Germany). Enzyme-linked immunosorbent assay (ELISA) kits for the determination of gonadotropin-releasing hormone (GnRH), follicle-stimulating hormone (FSH), luteinizing hormone (LH), progesterone (P_4_), and estradiol (E_2_) in ewes’ plasma were all purchased from Shanghai Enzyme-linked Biotechnology Co., Ltd. (Shanghai, China).

### 2.3. Experimental Design and Grouping

The trial was conducted from October 2024 to December 2024 at Huishang Ecological Animal Husbandry Co., Ltd. (Turpan, China) (coordinates 87°14′05″–89°11′08″ E, 41°21′14″–43°18′11″ N), with average outdoor and indoor temperatures of 35.5 °C and 38.8 °C, respectively.

Twelve non-pregnant multiparous Turpan black ewes selected and weighed at 18 months of age (51.65 kg ± 2.49 kg) were surgically fitted with permanent proximal duodenal cannulas. After wound healing, they were randomly divided into a control group (Con group) and a treatment group (L-Cit group), with 6 ewes per group, for a 30-day infusion trial. After an 8-day acclimatization period to allow the ewes to adapt to the handling and infusion procedures, on trial day 0, the Con group received a duodenal infusion of 100 mL physiological saline, while the L-Cit group received a duodenal infusion of 0.25 g/kg BW^−1^ L-Cit dissolved in 100 mL physiological saline. A modified infusion set (20 drops/mL) was used. The total infusion volume was 100 mL over 30 min, with an infusion rate set at 3.33 mL/min (66 drops/min). The flow rate was checked and corrected every 5 min to ensure stability. After 8 consecutive days of infusion, on day 8, total fecal and urine collection was conducted to determine nitrogen metabolism and amino acid content. Simultaneously, feces and blood were collected for microbial and reproductive hormone analysis, respectively. Blood samples were collected 2 h post-infusion for plasma metabolite analysis.

**Figure 1:**
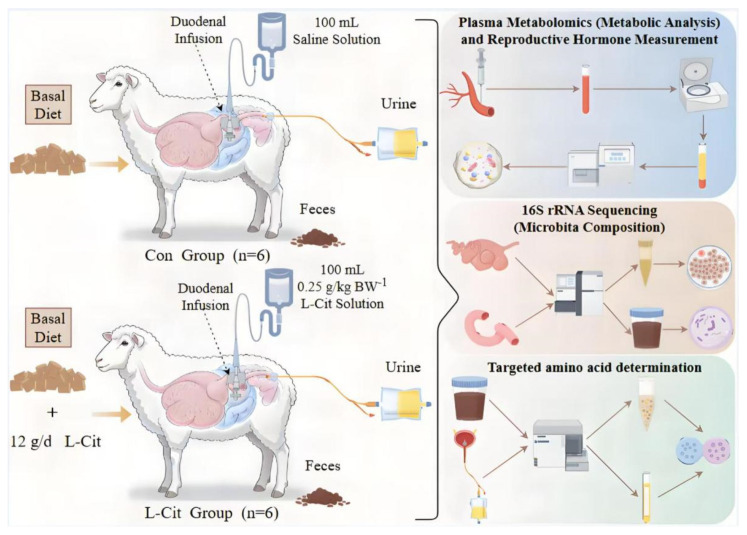
Experimental design diagram.

The L-Cit infusion and supplementation doses in this trial were based on studies by Gilbreath [14], Zhao [15], Ma [12], and An [16], who supplemented sheep with 10~12 g/d L-Cit. Studies have shown that in order to eliminate the effects of duodenal fluid load on digesta flow rate, digestive enzyme secretion, and intestinal absorption function, it is essential that the control group receive an equal volume of liquid (e.g., physiological saline) as the treatment group. Without such control, any observed changes in the experimental group (e.g., improvements in nitrogen metabolism) cannot be definitively attributed to the amino acids themselves, as opposed to being a result of altered intestinal osmotic pressure or physical stimulation caused by the additional fluid [17]. The specific experimental design flowchart is shown in Figure 1.

### 2.4. Animal Husbandry and Management

During the duodenal L-Cit infusion trial, all multiparous Turpan black ewes were individually housed and fed a TMR diet with consistent nutritional levels under identical conditions. They were fed twice daily at 10:00 and 18:00, and refusals were weighed and collected at 12:00 and 20:00. Each ewe had individual feed troughs and water buckets, ensuring ad libitum access to feed and water. The daily dry matter intake was approximately 1.21 kg/d per ewe. The TMR mixed feed was provided by Huishang Ecological Animal Husbandry Co., Ltd. All ewes in this trial were housed in individual pens, with twelve pens divided in the same penhouse. Each ewe was fed an equal amount of the same feed. Its composition and nutritional levels are shown in Table 1. The basal diet was not formulated to contain supplemental L-Cit, and any naturally occurring background levels were considered negligible and consistent across both groups.

### 2.5. Sample Collection

#### 2.5.1. Plasma Sample Collection and Processing

On day 8 of the formal feeding period, jugular venous blood was collected from ewes receiving duodenal L-Cit infusion at 0 h (pre-infusion) and 1, 2, 4, 6, and 8 h post-infusion. Blood was collected into heparinized tubes. For reproductive hormone assays, plasma was immediately separated by centrifugation at 2000× *g* (3500 rpm) for 15 min at 4 °C, aliquoted into 2 mL Eppendorf tubes, and stored at −20 °C. For plasma metabolome analysis, a dedicated blood sample collected at 2 h post-infusion was processed similarly: after collection into a heparinized tube, it was centrifuged at 2000× *g* (3500 rpm) for 15 min at 4 °C. The resulting plasma supernatant was aliquoted into labeled 2 mL Eppendorf tubes, rapidly frozen in liquid nitrogen, and stored at −80 °C.

#### 2.5.2. Feces and Urine Collection

On day 8 of continuous L-Cit infusion, total fecal and urine collection was conducted. Feces were collected and weighed before morning feeding daily during the formal period. A 10% subsample (by weight) was taken, mixed, and preserved with 10% sulfuric acid for nitrogen fixation. The treated feces were air-dried in a dry, ventilated area, weighed, ground, and passed through a 40-mesh sieve for amino acid and microbial analysis. Urine was collected twice daily, with the volume measured and recorded, and a 2% aliquot of the total volume was placed into a urine sample bottle containing 2 mL of 10% sulfuric acid and was stored at −20 °C for amino acid analysis.

### 2.6. Measurement Indicators

#### 2.6.1. Plasma Reproductive Hormones and Metabolites

Plasma levels of GnRH, FSH, LH, P_4_, and E_2_ were determined using enzyme-linked immunosorbent assay (ELISA) kits purchased from Shanghai Enzyme-linked Biotechnology Co., Ltd. The specific methods can be referred to in [18].

#### 2.6.2. Determination of Microorganisms in Feces

DNA was purified from feces and rumen fluid using the cetyltrimethylammonium bromide (CTAB) method and used as a template for PCR amplification with specific primers. PCR products were quantified by fluorescence (Qubit 3.0). Samples were sent to Shanghai Majorbio Bio-pharm Technology Co., Ltd. (Shanghai, China) for analysis. Specific methods can be referred to in [19].

#### 2.6.3. Targeted Amino Acid Analysis in Feces and Urine

Sample pre-treatment: (1) Extraction: Metabolites were extracted using a methanol/acetonitrile–water system (typically 4:1 ratio) combined with liquid nitrogen freezing–grinding. (2) Derivatization: For some difficult-to-ionize amino acids (e.g., glutamine, asparagine), AccQ-Tag^TM^ or dansyl chloride derivatization was used to enhance mass spectrometry sensitivity. (3) Purification: Proteins, lipids, and other interferents were removed by solid-phase extraction (SPE) or ultrafiltration. Key parameter optimization: (1) Chromatographic conditions: Reverse-phase column (e.g., ACQUITY UPLC HSS T3, 1.8 μm); (2) Mobile phase: 0.1% formic acid in water (A)–acetonitrile (B), gradient elution (20–40 min); (3) Mass spectrometry parameters: Electrospray ionization (ESI+/− switching), MRM mode acquisition, collision energy optimized for maximum fragment ion abundance. The detection method can be referred to in [20].

### 2.7. Statistical Analysis

Excel was used for the preliminary organization of crude protein content, nitrogen metabolism indicators and reproductive hormone data. Subsequently, SPSS 26.0 was used for one-way ANOVA, followed by Duncan’s multiple-comparison test to assess differences between groups.

The raw plasma metabolite data were processed using the metabolomics software Progenesis QI v3.0 (Waters Corporation, Milford, CT, USA) for peak extraction, alignment, identification, etc., resulting in a data matrix containing retention time, peak area, mass-to-charge ratio, and identification information for subsequent bioinformatics analysis, performed by Shanghai Majorbio Bio-pharm Technology Co., Ltd. (Shanghai, China).

For data collected over time (plasma reproductive hormones), normality was tested using the Shapiro–Wilk test, and homogeneity of variances was assessed with Levene’s test. These data were then analyzed using a repeated-measures ANOVA, with treatment as a between-subjects factor and time as a within-subjects factor. When a significant treatment × time interaction was detected, post hoc comparisons at individual time points were performed with a Bonferroni correction for multiple comparisons.

Origin software was used to analyze correlations between reproductive hormones, gastrointestinal microbiota, and plasma metabolites. Data visualization was performed using GraphPad Prism 9.0 and Adobe Illustrator 2025. Results are presented as mean ± standard error. *p* < 0.05 was considered statistically significant, and *p* < 0.01 was considered highly significant.

## 3. Results

### 3.1. Effects of Duodenal L-Cit Infusion on Plasma Metabolites in Ewes

#### 3.1.1. Venn Diagram of Plasma Metabolite Composition

**Figure 2:**
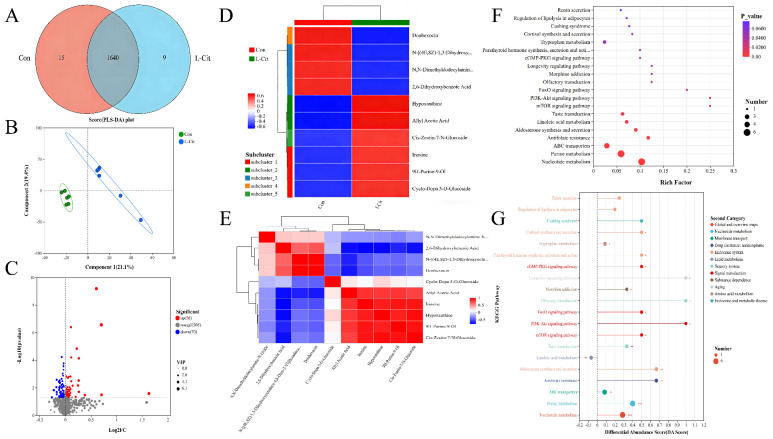
(**A**) Venn diagram of plasma metabolites; (**B**) PLS-DA score plot (Note: The x-axis represents the predicted principal component score for the first principal component, the y-axis represents the orthogonal principal component score; scatter shapes and colors represent different experimental groups); (**C**) volcano plot of differential plasma metabolites 2 h after duodenal L-Cit infusion; (**D**) hierarchical clustering heatmap of differential metabolites between groups; (**E**) correlation analysis plot of differential metabolites between groups; (**F**) KEGG enrichment analysis bubble plot (Note: The x-axis represents the impact value for each pathway, the y-axis represents pathway names; results are presented as a bubble plot; bubble color indicates the *p*-value of enrichment analysis, a redder color indicates more significant enrichment; bubble size represents the number of differential metabolites enriched in that pathway); (**G**) KEGG pathway differential abundance score plot (Note: The x-axis represents the differential abundance (DA) score, the y-axis represents KEGG pathway names; DA score reflects the overall change in all metabolites in a pathway, a score of 1 indicates up-regulation of all annotated differential metabolites in that pathway, −1 indicates down-regulation; line segment length represents the absolute value of DA score; dot size represents the number of annotated differential metabolites in that pathway, larger dots indicate more differential metabolites; dots on the right side of the central axis with longer lines indicate a tendency for overall up-regulation; dots on the left side with longer lines indicate a tendency for overall down-regulation).

As shown in Figure 2A, after processing the sequencing data and partitioning metabolites based on similarity, 24 distinct metabolites were identified. The Con group had 1655 metabolites, the L-Cit group had 1649 metabolites, and 1644 metabolites were common to both groups.

#### 3.1.2. Partial Least Squares-Discriminant Analysis (PLS-DA)

The results are shown in Figure 2B. The PLS-DA score plot visually displays the model’s classification effectiveness. Greater separation between the two groups indicates more significant classification. The first principal component explained 21.10% of the variance, and the second explained 19.40%.

#### 3.1.3. Screening of Differential Plasma Metabolites

The results are shown in Figure 2C. On day 4 of duodenal L-Cit infusion, 1632 differential metabolites were detected in ewes’ plasma between the Con and L-Cit groups, of which 126 showed significant changes (7.72% of total metabolites). Among these, 70 were down-regulated (4.29% of total, 55.56% of differential metabolites) and 56 were up-regulated (3.43% of total, 44.44% of differential metabolites) (*p* < 0.05). A total of 1506 metabolites showed no significant change.

#### 3.1.4. Cluster Analysis of Plasma Metabolites

The hierarchical clustering analysis heatmap results are shown in Figure 2D. In the Con group, N,N-Dimethyldodecylamine-N-Oxide increased, while Allyl Acetic Acid, Cyclo-Dopa 5-O-Glucoside, and Cis-Zeatin-7-N-Glucoside decreased. In the L-Cit group, 2,6-Dihydroxybenzoic Acid decreased.

#### 3.1.5. Correlation Analysis of Plasma Metabolites

As shown in Figure 2E, metabolite correlations often reveal synergistic changes: Cyclo-Dopa 5-O-Glucoside, Allyl Acetic Acid, inosine, Hypoxanthine, 9H-Purine-9-Ol, and Cis-Zeatin-7-N-Glucoside showed similar trends, indicating positive correlation. N,N-Dimethyldodecylamine-N-Oxide, 2,6-Dihydroxybenzoic Acid, N-[(4E,8Z)-1,3-Dihydroxyoctadeca-4,8-Dien-2-Yl]Hexadecanamide 1-Glucoside, and Donhexocin showed opposite trends, indicating negative correlation.

#### 3.1.6. KEGG Pathway Enrichment Analysis

As shown in Figure 2F, among the top 20 enriched pathways, 7 showed significant differences: nucleotide metabolism, purine metabolism, ABC transporters, Antifolate resistance, Aldosterone synthesis and secretion, linoleic acid metabolism, and Taste transduction. This indicates that most differential metabolites are closely related to the biological functions of these seven pathways.

#### 3.1.7. KEGG Pathway Differential Abundance Score

As shown in Figure 2G, purine metabolism and nucleotide metabolism showed significantly higher up-regulation trends than other pathways. One pathway, linoleic acid metabolism, showed a tendency for down-regulation.

### 3.2. Effects of Duodenal L-Cit Infusion on Nitrogen Metabolism and Amino Acid Content in Ewes

#### 3.2.1. Feed Intake, Water Intake, Total L-Cit Infusion, Total Fecal Output, and Total Urine Output

As shown in Table 2, compared to the Con group, the L-Cit group showed slightly higher feed and water intake, but the differences were not significant (*p* > 0.05). Total fecal and urine output were slightly lower in the L-Cit group, but the differences were not significant (*p* > 0.05).

#### 3.2.2. Effects of Duodenal L-Cit Infusion on Nitrogen Metabolism in Ewes

As shown in Table 3, crude protein (CP) content in feces was significantly lower by 11.96% (*p* < 0.05) and in urine it was extremely significantly lower by 34.20% (*p* < 0.01) in the L-Cit group compared to the Con group.

As shown in Table 4, total nitrogen intake was 24.35 g/d for both groups. Fecal nitrogen in the L-Cit group was 11.14% lower (difference 1.55 g/d), urinary nitrogen was 21.14% lower (difference 0.89 g/d), total excreted nitrogen was 12.97% lower (difference 2.35 g/d), retained nitrogen was 36.45% higher (difference 3.31 g/d), and nitrogen utilization rate was 50.15% higher in the L-Cit group. The biological value of nitrogen in the L-Cit group was 19.93% higher than in the Con group. However, none of these nitrogen metabolism indicators showed statistically significant differences between groups (*p* > 0.05).

The symbols a and b typically denote the 0.05 significance level, indicating that the probability of error is less than 5%; whereas A and B generally represent the 0.01 extremely significant level, meaning the probability of error is less than 1%.

#### 3.2.3. Effects of Duodenal L-Cit Infusion on Fecal Amino Acids in Ewes

**Figure 3:**
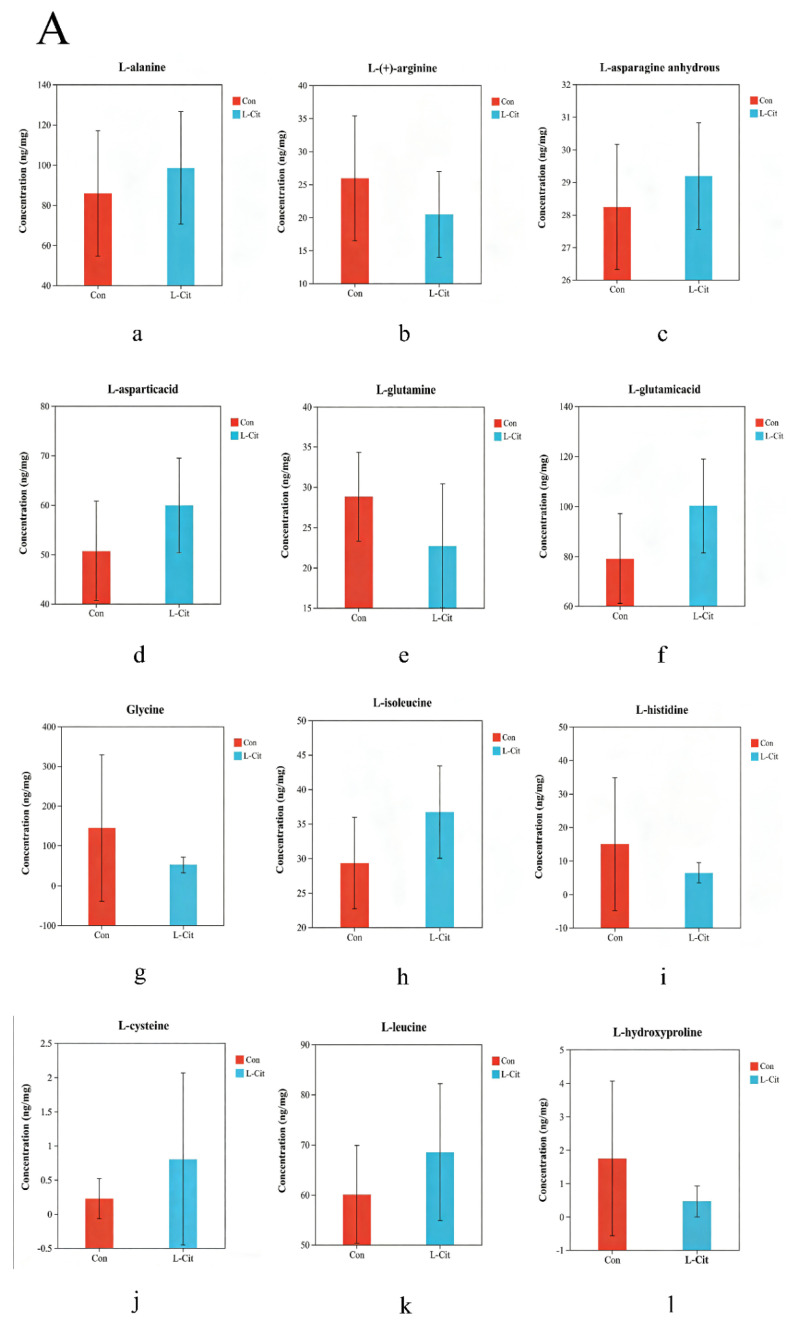

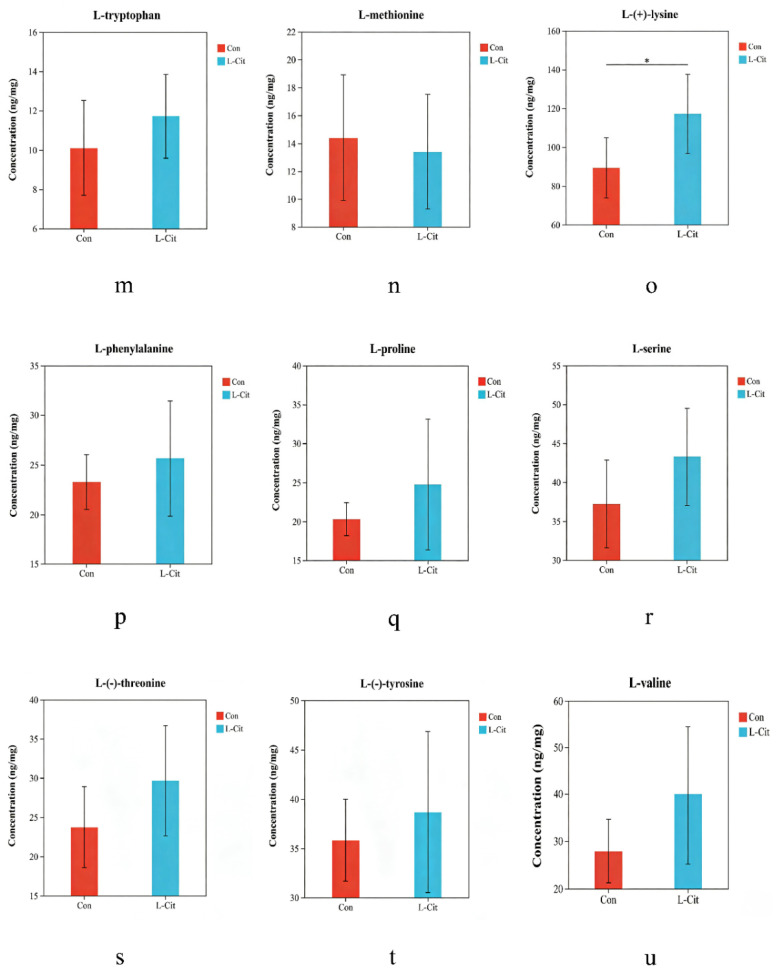

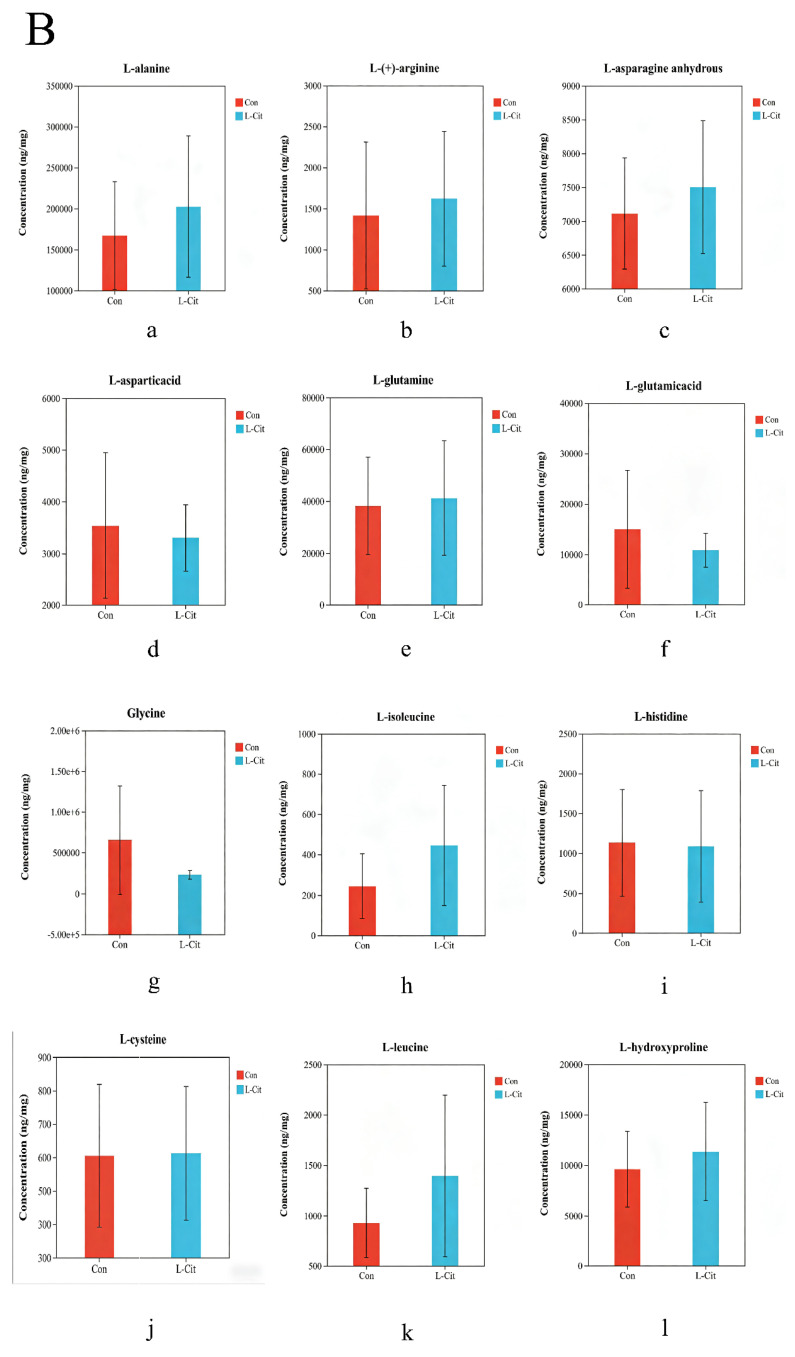

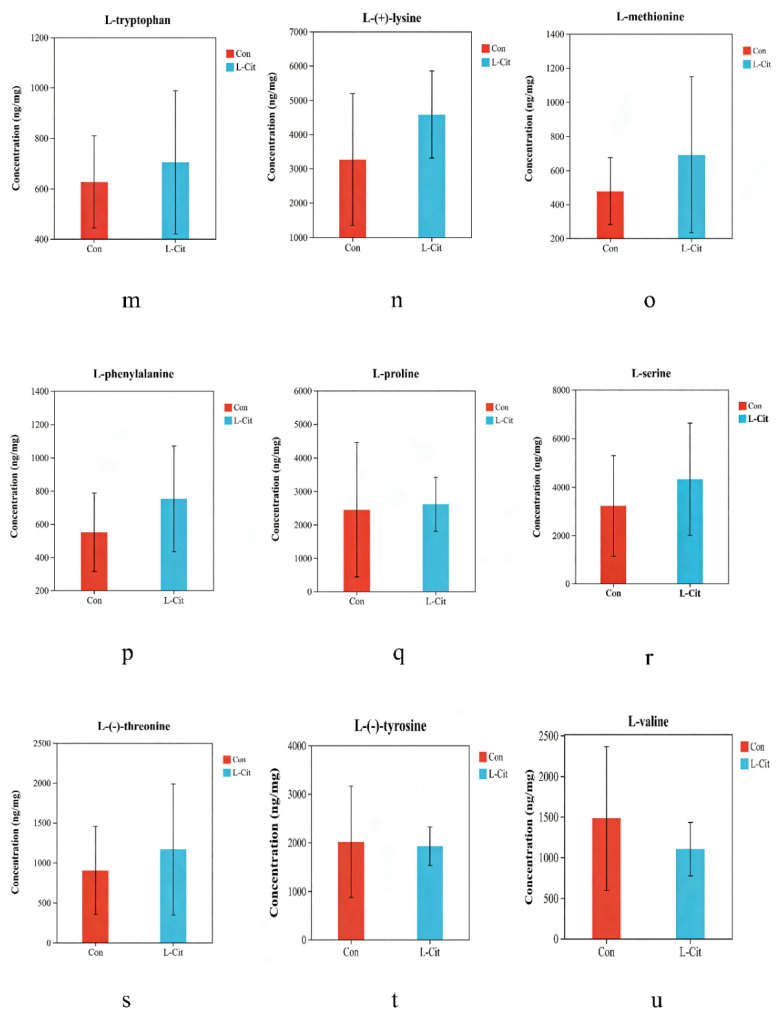
(**A**) Effects of duodenal L-Cit infusion on relative abundance of fecal amino acids in ewes. (**B**) Effects of duodenal L-Cit infusion on relative abundance of urinary amino acids in ewes. Note: In (**A**) (**a**): L-alanine; (**b**): L-asparagine anhydrous; (**c**): L-(+)-arginine; (**d**): L-aspartic acid; (**e**): L-glutamine; (**f**): L-glutamic acid; (**g**): glycine; (**h**): L-isoleucine; (**i**): L-histidine; (**j**): L-cysteine; (**k**): L-leucine; (**l**): L-hydroxyproline; (**m**): L-tryptophan; (**n**): L-methionine; (**o**): L-(+)-lysine; (**p**): L-phenylalanine; (**q**): L-proline; (**r**): L-serine; (**s**): L-(-)-threonine; (**t**): L-(-)-tyrosine; (**u**): L-valine. In (**B**) (**a**): L-alanine; (**b**): L-(+)-arginine; (**c**): L-asparagine anhydrous; (**d**): L-aspartic acid; (**e**): L-glutamine; (**f**): L-glutamic acid; (**g**): glycine; (**h**): L-isoleucine; (**i**): L-histidine; (**j**): L-cysteine; (**k**): L-leucine; (**l**): L-hydroxyproline; (**m**): L-tryptophan; (**n**): L-(+)-lysine; (**o**): L-methionine; (**p**): L-phenylalanine; (**q**): L-proline; (**r**): L-serine; (**s**): L-(-)-threonine; (**t**): L-(-)-tyrosine; (**u**): L-valine.

The effects of duodenal L-Cit infusion on fecal amino acid content are shown in Figure 3A(o). Compared to the Con group, L-lysine content in the L-Cit group increased significantly by 31.15% (*p* < 0.05). As shown in Figure 3A, levels of L-alanine, L-asparagine, L-aspartic acid, L-glutamic acid, L-isoleucine, L-cysteine, L-leucine, L-tryptophan, L-phenylalanine, L-proline, L-serine, L-threonine, L-tyrosine, and L-valine all increased; while levels of L-Arg, L-glutamine, glycine, L-histidine, L-hydroxyproline, and L-methionine all decreased. However, none of these changes were significant (*p* > 0.05).

#### 3.2.4. Effects of Duodenal L-Cit Infusion on Urinary Amino Acids in Ewes

The effects of duodenal L-Cit infusion on targeted urinary amino acid content are shown in Figure 3B. Compared to the Con group, the L-Cit group showed increased levels of L-alanine, L-Arg, L-asparagine, L-glutamine, L-isoleucine, L-cysteine, L-leucine, L-hydroxyproline, L-tryptophan, L-lysine, L-methionine, L-phenylalanine, L-proline, L-serine, and L-threonine; while levels of L-aspartic acid, L-glutamic acid, glycine, L-histidine, L-tyrosine, and L-valine decreased. None of these changes were significant (*p* > 0.05).

### 3.3. Effects of Duodenal L-Cit Infusion on Fecal Microbiota in Ewes

#### 3.3.1. Fecal Microbial Community Composition

**Figure 4:**
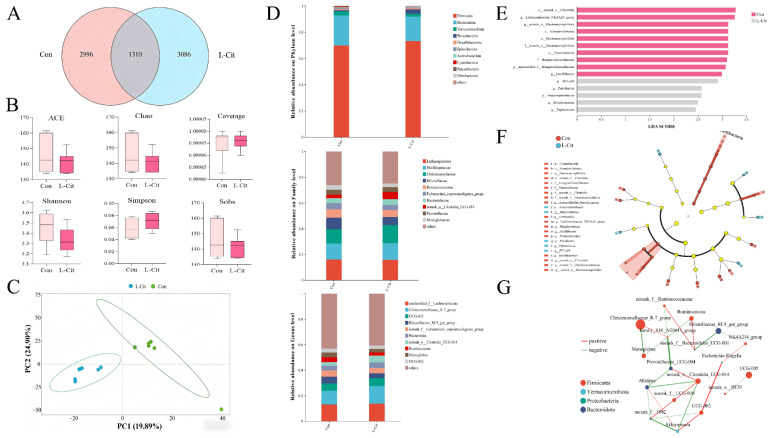
(**A**) Venn diagram of species/operational taxonomic unit (OTU) analysis. (**B**) Box plots of alpha diversity indices. (**C**) Partial least squares-discriminant analysis (PLS-DA) score plot (Note: The x-axis represents the predicted principal component score for the first principal component, and the y-axis represents the orthogonal principal component score. Scatter point shapes and colors denote different experimental groups). (**D**) Relative abundance of fecal microbiota at the phylum, family, and genus levels in ewes following duodenal L-Cit infusion (Note: The x-axis/y-axis represents sample names, and the y-axis/x-axis represents the proportion of a species within the corresponding sample. Bars of different colors represent different species, and bar length corresponds to the proportional abundance). (**E**) LEfSe (Linear Discriminant Analysis Effect Size) cladogram depicting the taxonomic hierarchy of fecal microbiota composition in ewes after duodenal L-Cit infusion (Note: The LDA discriminant bar chart identifies microbial taxa with significant differential abundance among multiple groups, based on Linear Discriminant Analysis). (**F**) Linear Discriminant Analysis (LDA) of fecal microbiota composition following duodenal L-Cit infusion in ewes (Note: The letters p, c, o, f, and g denote the taxonomic ranks of phylum, class, order, family, and genus, respectively). (**G**) Correlation network analysis of fecal microbiota composition after duodenal L-Cit infusion in ewes (Note: Node size represents species abundance. Species belonging to the same higher taxonomic level (e.g., genus) are uniformly colored, with different colors representing distinct higher taxonomic levels. Line color indicates positive (red) or negative (green) correlation. Line thickness corresponds to the magnitude of the correlation coefficient, with thicker lines indicating stronger correlations. A higher number of connecting lines signifies that a species is more closely associated with other species in the network).

After processing the sequencing data and clustering sequences at 97% similarity into OTUs, the results are shown in Figure 4A. A total of 7392 different OTUs were identified between the Con and L-Cit groups. The number of unique OTUs within the Con and L-Cit groups were 2996 and 3086, respectively. The Con group had a total of 4306 OTUs, the L-Cit group had 4396 OTUs, and 1310 OTUs were shared.

#### 3.3.2. Alpha Diversity Analysis

As shown in Figure 4B, no significant differences were observed across the indices between the Con group and the L-Cit group, indicating that duodenal infusion of L-Cit did not exert a significant impact on the diversity of the fecal microbial community in ewes. Furthermore, the species coverage for all samples reached 99.9%, demonstrating that the sequencing depth was sufficient to adequately represent the true composition of the microbial community in the fecal samples.

#### 3.3.3. PLS-DA Analysis

As shown in Figure 4C, the first principal component contributed 19.89% to sample variation, and the second contributed 24.90%. The Con and L-Cit groups were separated into two clusters. The Con group’s microbial composition was more compact with smaller community differences, while the L-Cit group’s composition was more dispersed with larger community differences.

#### 3.3.4. Effects of Duodenal L-Cit Infusion on Fecal Microbial Species Composition and Differential Analysis

##### Effects at the Phylum Level

The composition of fecal microbiota in Con and L-Cit groups after duodenal L-Cit infusion is shown in Figure 4D. At the phylum level, major phyla included *Firmicutes* (69.91%; 73.22%), *Bacteroidetes* (22.87%; 18.55%), *Verrucomicrobia* (3.02%; 3.31%), *Proteobacteria* (0.98%; 2.23%), *Desulfobacterota* (0.86%; 1.03%), *Spirochaetota* (0.76%; 0.45%), *Actinobacteriota* (0.67%; 0.52%), *Cyanobacteria* (0.46%; 0.19%), *Patescibacteria* (0.28%; 0.15%), and *Fibrobacterota* (0.04%; 0.20%). The Con group had higher relative abundances of *Bacteroidetes* and *Cyanobacteria*, while the L-Cit group had higher abundances of *Firmicutes*, *Proteobacteria*, and *Fibrobacterota*.

##### Effects at the Family Level

As shown in Figure 4D, at the family level, major families included *Lachnospiraceae* (15.87%; 15.66%), *Oscillospiraceae* (12.59%; 12.91%), *Christensenellaceae* (11.08%; 14.05%), *Rikenellaceae* (9.04%; 6.33%), *Ruminococcaceae* (6.53%; 5.56%), *Bacteroidaceae* (3.88%; 4.41%), *Prevotellaceae* (3.75%; 3.87%), and *Monoglobaceae* (3.67%; 2.70%). The Con group had a higher relative abundance of *Rikenellaceae*, while the L-Cit group had a higher abundance of *Christensenellaceae*.

##### Effects at the Genus Level

As shown in Figure 4D, at the genus level, enrichment was observed in genera such as *Christensenellaceae_R-7_group* (10.78%; 13.92%), *UCG-005* (5.46%; 6.06%), *Rikenellaceae_RC9_gut_group* (5.54%; 3.81%), *Eubacterium_coprostanoligenes_group* (4.74%; 4.05%), *Bacteroides* (3.88%; 4.41%), *Ruminococcus* (3.90%; 0%), *Monoglobus* (3.67%; 2.70%), and *UCG-002* (2.97%; 2.62%). The Con group showed enrichment in *Ruminococcus*.

#### 3.3.5. Differential Analysis of Fecal Microbial Species Composition

Based on the analyses of community structure and species composition described above, which indicated that duodenal infusion of L-Cit induced certain alterations in the fecal microbiota of ewes, we further employed LEfSe (Linear Discriminant Analysis Effect Size) to identify microbial taxa with significantly differential relative abundance between the two groups. The analysis was conducted using an LDA (Linear Discriminant Analysis) effect size threshold of >2 and a significance level of *p* < 0.05. The results are presented in Figure 4E.

As shown in Figure 4F, at the phylum level, the relative abundance of Cyanobacteria was significantly higher in the Con group (LDA = 3.12, *p* = 0.037). At the family level, *Hungateiclostridiaceae* was significantly higher in the Con group (LDA = 3.07, *p* = 0.037), while *Planococcaceae* was significantly higher in the L-Cit group (LDA = 3.07, *p* = 0.046).

At the genus level, relative abundances of *Lachnospiraceae_NK3A20_group* (LDA = 3.27, *p* = 0.016), *Oscillibacter* (LDA = 2.98, *p* = 0.025), and *Mogibacterium* (LDA = 2.89, *p* = 0.006) were significantly higher in the Con group. In contrast, *SP3-e08* (LDA = 2.93, *p* = 0.022), *Parvibacter* (LDA = 2.58, *p* = 0.022), *Anaerosporobacter* (LDA = 2.54, *p* = 0.022), *Butyricimonas* (LDA = 2.51, *p* = 0.033), and *Peptococcus* (LDA = 2.48, *p* = 0.009) were significantly higher in the L-Cit group (LDA > 2, *p* < 0.05).

#### 3.3.6. Fecal Microbiota Correlation Analysis

As shown in Figure 4G, Spearman correlation analysis indicated that six genera were interconnected: *Lachnospiraceae*, *Clostridia_UCG-014*, *Prevotellaceae_UCG-004*, *F082*, *Akkermansia*, and *Alistipes*. *Monoglobus* showed high correlation with *Christensenellaceae_R-7_group*. *Rikenellaceae_RC9_gut_group* showed positive correlation with *unclassified_c_Clostridia* and negative correlation with *NK4A214_group_UCG-002*.

### 3.4. Effects of Duodenal L-Cit Infusion on Plasma Reproductive Hormone Levels in Ewes

**Figure 5:**
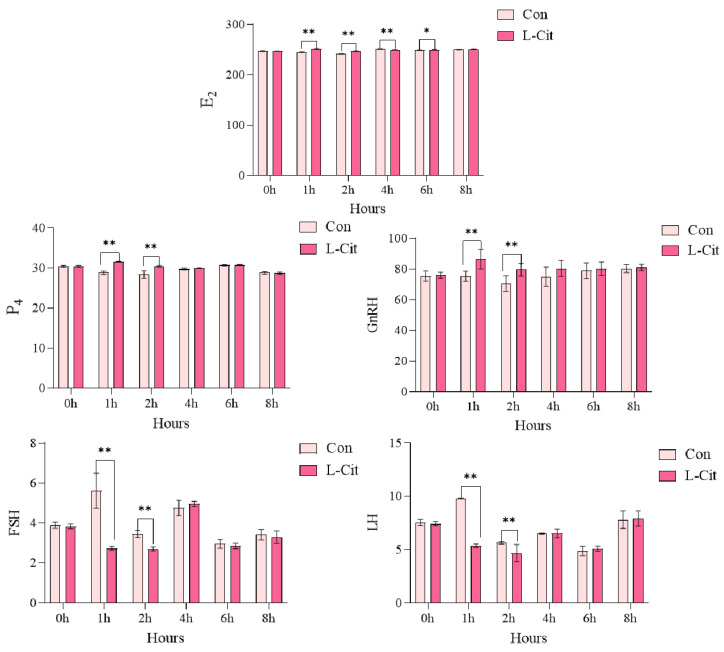
Effects of duodenal L-Cit infusion on plasma reproductive hormone levels in ewes. Note: E_2_ (pg/mL); P_4_ (ng/mL); GnRH (pg/mL); FSH (ng/mL); LH (pg/mL); “*” indicates significant difference, “**” indicates highly significant difference. The reported *p*-values for hormone changes at 1 h and 2 h are derived from the repeated-measures analysis.

As shown in Figure 5, compared to the Con group at 1 h post-infusion, the L-Cit group showed an extremely significant decrease in E_2_ by 2.58% (*p* < 0.01) and extremely significant increases in P_4_ by 9.73% (*p* < 0.01), GnRH by 14.95% (*p* < 0.01), FSH by 51.34% (*p* < 0.01), and LH by 45.25% (*p* < 0.01).

At 2 h, compared to the Con group, the L-Cit group showed an extremely significant decrease in E_2_ by 2.06% (*p* < 0.01) and extremely significant increases in P_4_ by 7.12% (*p* < 0.01), GnRH by 13.01% (*p* < 0.01), FSH by 22.03% (*p* < 0.01), and LH by 17.23% (*p* < 0.01).

At 4 h, compared to the Con group, E_2_ in the L-Cit group decreased extremely significantly by 0.55% (*p* < 0.01). At 6 h, E_2_ decreased significantly by 0.42% (*p* < 0.05).

No significant differences in E_2_ were observed at 0 h and 8 h. No significant differences in P_4_, GnRH, FSH, and LH were observed at 0 h, 4 h, 6 h, and 8 h.

The reported *p*-values for hormone changes at 1 h and 2 h are derived from the repeated-measures analysis.

## 4. Correlation Analysis

### 4.1. Correlation Analysis Between Fecal Microbiota and Plasma Metabolites in Ewes After L-Cit Infusion

#### 4.1.1. Correlation Analysis at the Phylum Level

**Figure 6:**
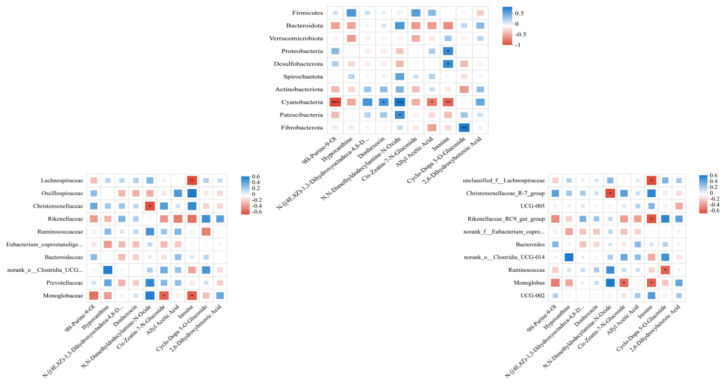
Heatmap of correlation analysis results between fecal microbiota (phylum, family, genus levels) and plasma metabolites. Note: The x-axis and y-axis represent plasma metabolites and fecal microbial species, respectively. R values and *p* values were calculated. R values are displayed in different colors in the figure, and *p* values are marked with an asterisk (*) if <0.05. The legend on the right shows the color ranges for different R values: * 0.01 < *p* ≤ 0.05, ** 0.001 < *p* ≤ 0.01, and *** *p* ≤ 0.001.

As shown in Figure 6, correlation analysis between fecal microbiota and plasma metabolites at the phylum level revealed that *Proteobacteria* and *Desulfobacterota* abundances were significantly negatively correlated (0.01 < *p* ≤ 0.05) with inosine. *Cyanobacteria* abundance was extremely significantly negatively correlated (*p* ≤ 0.001) with 9H-Purine-9-Ol, extremely significantly negatively correlated (0.001 < *p* ≤ 0.01) with inosine, significantly positively correlated (0.01 < *p* ≤ 0.05) with Allyl Acetic Acid, extremely significantly negatively correlated (0.001 < *p* ≤ 0.01) with N,N-Dimethyldodecylamine-N-Oxide, and significantly negatively correlated (0.01 < *p* ≤ 0.05) with Donhexocin. *Patescibacteria* was significantly negatively correlated (0.01 < *p* ≤ 0.05) with N,N-Dimethyldodecylamine-N-Oxide. *Fibrobacterota* was extremely significantly negatively correlated (0.001 < *p* ≤ 0.01) with Cyclo-Dopa 5-O-Glucoside.

#### 4.1.2. Correlation Analysis at the Family Level

As shown in Figure 6, correlation analysis between fecal microbiota and plasma metabolites at the family level revealed that Lachnospiraceae abundance was significantly positively correlated (0.01 < *p* ≤ 0.05) with inosine. *Christensenellaceae* abundance was significantly positively correlated (0.01 < *p* ≤ 0.05) with N,N-Dimethyldodecylamine-N-Oxide. *Monoglobaceae* abundance was significantly negatively correlated (0.01 < *p* ≤ 0.05) with Cis-Zeatin-7-N-Glucoside and inosine.

#### 4.1.3. Correlation Analysis at the Genus Level

As shown in Figure 6, correlation analysis between fecal microbiota and plasma metabolites at the genus level revealed that *unclassified_f_Lachnospiraceae* abundance was significantly positively correlated (0.01 < *p* ≤ 0.05) with inosine. *Christensenellaceae_R-7_group* abundance was significantly positively correlated (0.01 < *p* ≤ 0.05) with N,N-Dimethyldodecylamine-N-Oxide. *Rikenellaceae_RC9_gut_group* abundance was significantly positively correlated (0.01 < *p* ≤ 0.05) with inosine. Ruminococcus abundance was significantly positively correlated (0.01 < *p* ≤ 0.05) with Cyclo-Dopa 5-O-Glucoside. *Monoglobus* abundance was significantly positively correlated (0.01 < *p* ≤ 0.05) with Cis-Zeatin-7-N-Glucoside and inosine.

### 4.2. Correlation Analysis Between Fecal Microbiota and Fecal Amino Acid Content in Ewes After L-Cit Infusion

#### 4.2.1. Correlation Analysis at the Phylum Level

**Figure 7:**
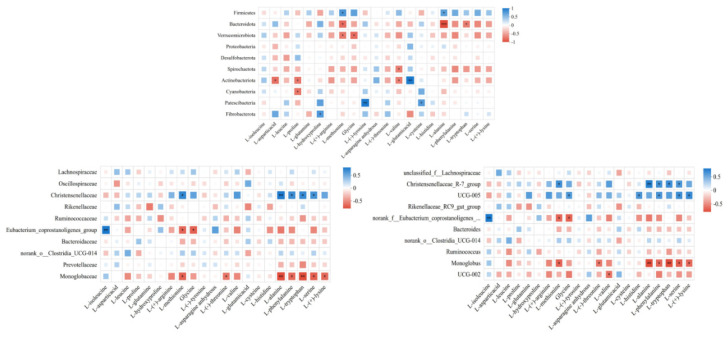
Heatmap of correlation analysis results between fecal microbiota (phylum, family, genus levels) and fecal amino acid content. Note: The x-axis and y-axis represent fecal amino acid content and fecal microbial species, respectively. The R-value and *p*-value were calculated. The R-value is displayed in different colors in the figure, and *p*-values less than 0.05 are marked with an asterisk (*). The legend on the right shows the color ranges for different R-values: * 0.01 < *p* ≤ 0.05, ** 0.001 < *p* ≤ 0.01, and *** *p* ≤ 0.001.

As shown in Figure 7, correlation analysis between fecal microbiota and fecal amino acid content at the phylum level revealed that *Firmicutes* abundance was significantly negatively correlated (0.01 < *p* ≤ 0.05) with L-methionine and L-alanine. *Bacteroidota* abundance was extremely significantly positively correlated (*p* ≤ 0.001) with L-alanine and significantly positively correlated (0.01 < *p* ≤ 0.05) with L-methionine and L-tryptophan. *Verrucomicrobia* abundance was significantly positively correlated (0.01 < *p* ≤ 0.05) with L-methionine and glycine. *Spirochaetota* abundance was significantly positively correlated (0.01 < *p* ≤ 0.05) with L-valine. *Actinobacteriota* abundance was significantly positively correlated (0.01 < *p* ≤ 0.05) with L-asparagine anhydrous, L-proline, and L-valine and extremely significantly negatively correlated (0.001 < *p* ≤ 0.01) with L-glutamic acid. *Cyanobacteria* was significantly positively correlated (0.01 < *p* ≤ 0.05) with L-proline. *Patescibacteria* was extremely significantly negatively correlated (0.001 < *p* ≤ 0.01) with L-(-)-tyrosine and significantly negatively correlated (0.01 < *p* ≤ 0.05) with L-cysteine. *Fibrobacterota* was significantly negatively correlated (0.01 < *p* ≤ 0.05) with L-hydroxyproline.

#### 4.2.2. Correlation Analysis at the Family Level

As shown in Figure 7, correlation analysis between fecal microbiota and fecal amino acid content at the family level revealed that *Christensenellaceae* abundance was extremely significantly negatively correlated (0.001 < *p* ≤ 0.01) with L-alanine and significantly negatively correlated (0.01 < *p* ≤ 0.05) with L-methionine, L-phenylalanine, L-tryptophan, and L-serine. *Eubacterium_coprostanoligenes_group* abundance was significantly positively correlated (0.01 < *p* ≤ 0.05) with L-methionine and glycine and extremely significantly negatively correlated (0.001 < *p* ≤ 0.01) with L-isoleucine. *Monoglobaceae* abundance was extremely significantly positively correlated (0.001 < *p* ≤ 0.01) with L-alanine and L-tryptophan and significantly positively correlated (0.01 < *p* ≤ 0.05) with L-methionine, L-phenylalanine, L-serine, and L-(+)-lysine.

#### 4.2.3. Correlation Analysis at the Genus Level

As shown in Figure 7, correlation analysis between fecal microbiota and fecal amino acid content at the genus level revealed that *Christensenellaceae_R-7_group* abundance was extremely significantly negatively correlated (0.001 < *p* ≤ 0.01) with L-alanine and significantly negatively correlated (0.01 < *p* ≤ 0.05) with L-methionine, L-phenylalanine, L-tryptophan, and L-serine. UCG-005 abundance was significantly negatively correlated (0.01 < *p* ≤ 0.05) with glycine, L-histidine, L-phenylalanine, L-serine, and L-(+)-lysine. *Eubacterium_coprostanoligenes_group* abundance was significantly positively correlated (0.01 < *p* ≤ 0.05) with L-methionine and glycine and extremely significantly negatively correlated (0.001 < *p* ≤ 0.01) with L-isoleucine. *Monoglobus* abundance was extremely significantly positively correlated (0.001 < *p* ≤ 0.01) with L-alanine and L-tryptophan and significantly positively correlated (0.01 < *p* ≤ 0.05) with L-methionine, L-(-)-threonine, L-phenylalanine, and L-(+)-lysine. *UCG-002* was significantly positively correlated (0.01 < *p* ≤ 0.05) with L-valine.

### 4.3. Correlation Analysis Between Fecal Microbiota and Urinary Amino Acid Content in Ewes After L-Cit Infusion

#### 4.3.1. Correlation Analysis at the Phylum Level

**Figure 8:**
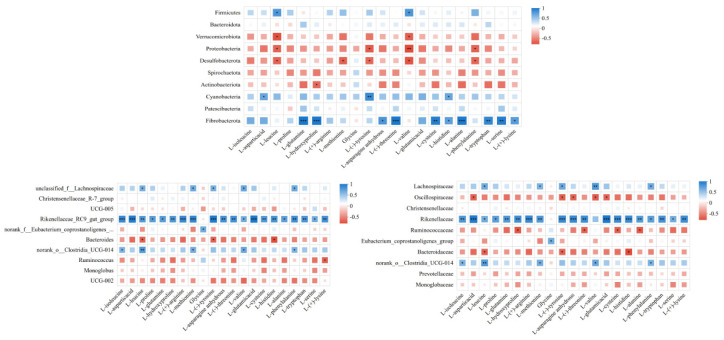
Heatmap of correlation analysis results between fecal microbiota (phylum, family, genus levels) and urinary amino acid content. Note: The x-axis and y-axis represent urinary amino acid content and fecal microbial species, respectively. The R value and *p* value were calculated. The R value is displayed in different colors in the figure, and a * symbol is used to indicate a *p* value <0.05. The legend on the right shows the color ranges for different R values: * 0.01 < *p* ≤ 0.05, ** 0.001 < *p* ≤ 0.01, and *** *p* ≤ 0.001.

As shown in Figure 8, correlation analysis between fecal microbiota and urinary amino acid content at the phylum level revealed that *Firmicutes* abundance was significantly negatively correlated (0.01 < *p* ≤ 0.05) with L-aspartic acid and L-valine. *Verrucomicrobia* abundance was significantly positively correlated (0.01 < *p* ≤ 0.05) with L-leucine and L-valine. *Proteobacteria* abundance was extremely significantly positively correlated (0.001 < *p* ≤ 0.01) with L-valine and significantly positively correlated (0.01 < *p* ≤ 0.05) with L-leucine, L-(-)-tyrosine, and L-phenylalanine. *Desulfobacterota* abundance was significantly positively correlated (0.01 < *p* ≤ 0.05) with L-leucine, L-methionine, L-(-)-tyrosine, L-valine, and L-phenylalanine. *Actinobacteriota* abundance was significantly positively correlated (0.01 < *p* ≤ 0.05) with L-hydroxyproline. *Cyanobacteria* abundance was extremely significantly negatively correlated (0.001 < *p* ≤ 0.01) with L-(-)-tyrosine and significantly negatively correlated (0.01 < *p* ≤ 0.05) with L-aspartic acid. *Fibrobacterota* abundance was extremely significantly negatively correlated (*p* ≤ 0.001) with L-glutamine, L-hydroxyproline, L-(-)-threonine, and L-alanine; extremely significantly negatively correlated (0.001 < *p* ≤ 0.01) with L-cysteine, L-tryptophan, and L-serine; and significantly negatively correlated (0.01 < *p* ≤ 0.05) with L-asparagine anhydrous, L-histidine, and L-(+)-lysine.

#### 4.3.2. Correlation Analysis at the Family Level

As shown in Figure 8, correlation analysis between fecal microbiota and urinary amino acid content at the family level revealed that *Lachnospiraceae* abundance was extremely significantly negatively correlated (0.001 < *p* ≤ 0.01) with L-valine and significantly negatively correlated (0.01 < *p* ≤ 0.05) with L-leucine, L-methionine, L-(-)-tyrosine, and L-phenylalanine. *Oscillospiraceae* abundance was significantly positively correlated (0.01 < *p* ≤ 0.05) with L-aspartic acid, L-(-)-tyrosine, L-asparagine anhydrous, L-valine, and L-glutamic acid. *Rikenellaceae* abundance was extremely significantly negatively correlated (*p* ≤ 0.001) with L-aspartic acid, L-(+)-arginine, L-(-)-tyrosine, L-asparagine anhydrous, L-glutamine, L-cysteine, and L-histidine; extremely significantly negatively correlated (0.001 < *p* ≤ 0.01) with L-isoleucine, L-glutamine, L-hydroxyproline, L-methionine, L-(-)-threonine, L-alanine, L-tryptophan, and L-(+)-lysine; and significantly negatively correlated (0.01 < *p* ≤ 0.05) with L-leucine, L-proline, L-phenylalanine, and L-serine. *Ruminococcaceae* abundance was significantly positively correlated (0.01 < *p* ≤ 0.05) with L-hydroxyproline, L-(-)-threonine, L-cysteine, and L-alanine. *Eubacterium_coprostanoligenes_group* abundance was significantly negatively correlated (0.01 < *p* ≤ 0.05) with glycine. *Bacteroidaceae* abundance was significantly positively correlated (0.01 < *p* ≤ 0.05) with L-leucine, L-(-)-tyrosine, and L-histidine. *norank_o_Clostridia_UCG-014* abundance was extremely significantly negatively correlated (0.001 < *p* ≤ 0.01) with L-leucine and significantly positively correlated (0.01 < *p* ≤ 0.05) with L-isoleucine, L-methionine, L-valine, and L-phenylalanine.

#### 4.3.3. Correlation Analysis at the Genus Level

As shown in Figure 8, correlation analysis between fecal microbiota and urinary amino acid content at the genus level revealed that *unclassified_f_Lachnospiraceae* abundance was significantly negatively correlated (0.01 < *p* ≤ 0.05) with L-leucine, L-methionine, L-(-)-tyrosine, L-valine, and L-phenylalanine. *Rikenellaceae_RC9_gut_group* abundance was extremely significantly negatively correlated (*p* ≤ 0.001) with L-isoleucine, L-aspartic acid, L-(+)-arginine, L-methionine, L-(-)-tyrosine, and L-glutamine; extremely significantly negatively correlated (0.001 < *p* ≤ 0.01) with L-leucine, L-hydroxyproline, L-asparagine anhydrous, L-(-)-threonine, L-cysteine, L-histidine, L-phenylalanine, L-tryptophan, and L-(+)-lysine; and significantly negatively correlated (0.01 < *p* ≤ 0.05) with L-proline, L-glutamine, L-valine, L-alanine, and L-serine. *norank_f_Eubacterium_coprostanoligenes_group* abundance was significantly negatively correlated (0.01 < *p* ≤ 0.05) with glycine. *Bacteroides* abundance was significantly positively correlated (0.01 < *p* ≤ 0.05) with L-leucine, L-(-)-tyrosine, and L-histidine. *norank_o_Clostridia_UCG-014* abundance was extremely significantly negatively correlated (0.001 < *p* ≤ 0.01) with L-leucine and significantly positively correlated (0.01 < *p* ≤ 0.05) with L-isoleucine, L-methionine, L-valine, and L-phenylalanine. *Ruminococcus* abundance was significantly positively correlated (0.01 < *p* ≤ 0.05) with L-(+)-lysine.

### 4.4. Correlation Analysis Between Fecal Microbiota and Plasma Reproductive Hormones in Ewes After L-Cit Infusion

#### 4.4.1. Correlation Analysis at the Phylum Level

**Figure 9:**
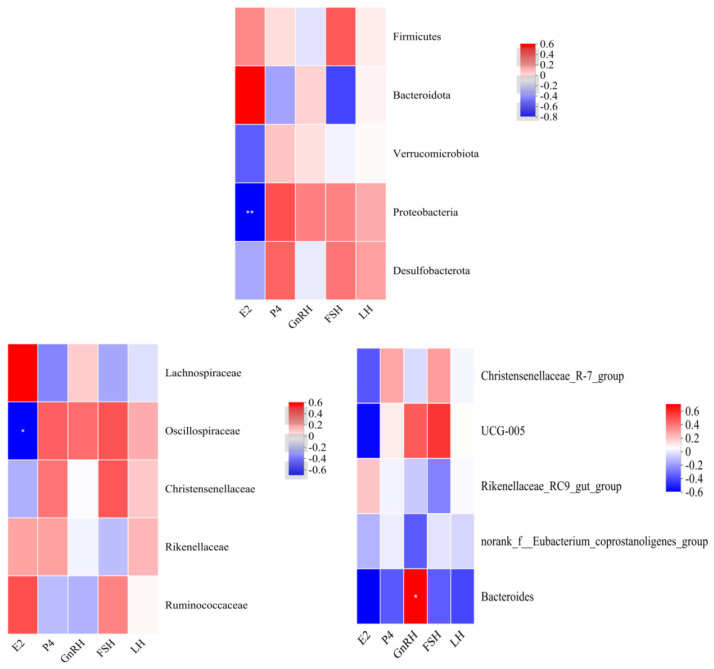
Heatmap of correlation analysis results between fecal microbiota (phylum, family, genus levels) and plasma reproductive hormones. Note: The x-axis and y-axis represent plasma reproductive hormones and fecal microbial species, respectively. R values and *p* values were calculated. R values are displayed in different colors in the figure, and *p* values less than 0.05 are marked with an asterisk (*). The legend on the right shows the color ranges for different R values: * 0.01 < *p* ≤ 0.05, ** 0.001 < *p* ≤ 0.01.

As shown in Figure 9, correlation analysis between fecal microbiota and plasma reproductive hormones at the phylum level revealed that E_2_ content was extremely significantly negatively correlated (0.001 < *p* ≤ 0.01) with *Proteobacteria* abundance.

#### 4.4.2. Correlation Analysis at the Family Level

As shown in Figure 9, correlation analysis between fecal microbiota and plasma reproductive hormones at the family level revealed that E_2_ content was significantly negatively correlated (0.01 < *p* ≤ 0.05) with *Oscillospiraceae* abundance.

#### 4.4.3. Correlation Analysis at the Genus Level

As shown in Figure 9, correlation analysis between fecal microbiota and plasma reproductive hormones at the genus level revealed that GnRH content was significantly positively correlated (0.01 < *p* ≤ 0.05) with *Bacteroidaceae* abundance.

### 4.5. Correlation Analysis Between Plasma Metabolites and Plasma Reproductive Hormones in Ewes After L-Cit Infusion

**Figure 10:**
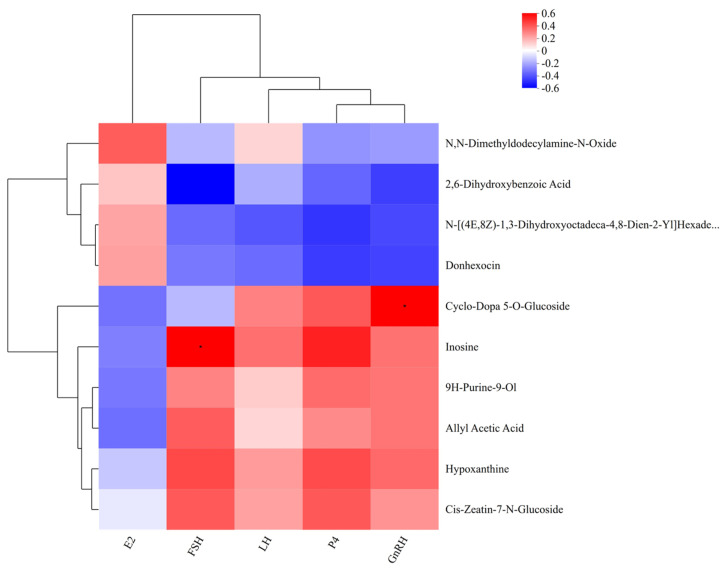
Heatmap of correlation analysis results between plasma metabolites and plasma reproductive hormones. Note: The x-axis and y-axis represent plasma reproductive hormones and plasma metabolites, respectively. R values and *p* values were calculated. R values are displayed in different colors in the figure, and *p* values are marked with an asterisk (*) if <0.05. The legend on the right shows the color ranges for different R values: * 0.01 < *p* ≤ 0.05.

As shown in Figure 10, correlation analysis between plasma metabolites and reproductive hormones revealed that E_2_ content was significantly positively correlated (0.01 < *p* ≤ 0.05) with 1-Aminocyclohexanecarboxylic Acid. FSH content was significantly positively correlated (0.01 < *p* ≤ 0.05) with inosine, significantly negatively correlated (0.001 < *p* ≤ 0.01) with Ile-Ile-Ile-Pro, and significantly negatively correlated (0.01 < *p* ≤ 0.05) with Asn-Gln-Lys. GnRH content was significantly negatively correlated (0.01 < *p* ≤ 0.05) with 8-Hydroxyquinoline and Cyclo-Dopa 5-O-Glucoside.

## 5. Discussion

### 5.1. Effects of Duodenal Infusion of L-Cit on Intestinal Absorption in Ewes

#### 5.1.1. Effects of Duodenal Infusion of L-Cit on Nitrogen Metabolism in Ewes

L-Citrulline (L-Cit) is used in the synthesis of L-arginine (L-Arg) and this synthesis mainly occurs in the small intestine. When taken in, L-Cit is transformed into L-Arg through the catalytic effect of argininosuccinate synthetase and lyase [4]. This metabolic pathway is characterized by the production of N-acetyl-L-glutamate using an acetyl-CoA and glutamate reaction that is assisted by N-acetylglutamate kinase. N-acetyl-L-glutamate is an allosteric activator of carbamoyl phosphate synthetase in mammals and stimulates the production of the L-Arg biosynthesis [21]. The production of L-Cit is finished by using ornithine transcarbamylase on carbamoyl phosphate and ornithine [22,23]. As a result, N-acetyl-L-glutamate is a very important cofactor to carbamyl phosphate synthetase in the urea pathway. In vivo, L-Arg has been metabolized to produce urea and NO [24] and thus controls the L-Arg-NO metabolic axis. The L-Cit-NO process is intracellular, which maintains a dynamic balance between L-Cit and L-Arg [25]. Intestinal L-Cit synthesis is important in endogenous L-Arg production. Inhibition of critical enzymes or small-intestinal resection may prevent the production of L-Cit in the intestine hence leading to L-Arg deficiency [26]. The results of the present research suggest that duodenal infusion with L-Cit can lead to an increased metabolism of NO, which could result in an increase in nutrient delivery in the organism [27]. Moreover, NO has been known to control the operations of the mitochondria, which in turn can indirectly enhance the effectiveness of protein synthesis and the digestion, absorption and metabolism of ingested protein in ruminant excretion. The protein consumed by ruminants is absorbed and digested and then metabolized into fecal and urinary nitrogen. Since the digestible fraction of nitrogen includes nitrogen expenditure in the form of urinary acid, nitrogen metabolism becomes a prime and straightforward measure of how an animal utilizes mineral nitrogen in its diet [28].

Although these improvements were numerically substantial, they did not reach statistical significance, likely due to the small sample size (*n* = 6) characteristic of this type of invasive physiological study, which limits statistical power and warrants cautious interpretation of the results.

Economical use of nitrogen will support maximum production in animals, especially in ruminants, wherein the process of microbial protein synthesis is of considerable importance. The results of this research show that the L-Cit duodenal infusion procedure succeeds in increasing levels of nitrogen retention and maximizing the efficiency of nitrogen usage by ewes. L-Cit is an essential intermediate in the urea cycle and its metabolite L-Arg is a very significant point in the detoxification process, as it transforms ammonia into urea [29]. It was found that L-Cit supplementation can optimize ammonia nitrogen recycling and thus minimize losses of nitrogen in the form of urea [30]. The surplus in the amount of crude protein cannot be stored and thus it has to be used by the kidneys. Feeding of high-protein diets over an extended period of time heightens the renal load to livestock since ammonia produced by protein breakdown has to be excreted in the urea cycle [31]. Protein metabolism causes ammonia, which is a toxic byproduct, to be removed by the promotion of urea cycle, which in turn decreases the excretion of nitrogen. This increased ability to retain nitrogen is central to maximization of protein synthesis and deposition capacity, which are critical in the reproductive system, such as in the growth of the follicles and uterus and implantation of embryos [32]. Empirical results declare that the supplementation with 12 g/d/head L-Cit increases the nitrogen retention and protein deposition of growing pigs [33]. In line with previous results, Lacroix [34] showed that L-Cit supplement enhances nitrogen absorption and milk protein synthesis in dairy cows. A recent experiment conducted on ewes found L-Cit duodenal infusion was highly effective in reducing the crude protein content in feces and urine together with fecal nitrogen, urinary nitrogen and total nitrogen excretion. At the same time, it was accompanied by a higher retained nitrogen level, rate of nitrogen utilization, and biological nitrogen value. These results support the fact that L-Cit is capable of increasing nitrogen metabolism in ewes to a large extent.

Although the numerical improvements in nitrogen retention, utilization rate, and biological value were substantial (36.45%, 50.15%, and 19.93% higher, respectively), none of these key indicators reached statistical significance (*p* > 0.05). This lack of significance is likely attributable to the small sample size (*n* = 6) characteristic of this invasive physiological model, which limits statistical power. This small sample size may not have been sufficient to detect medium-sized effects as statistically significant. Therefore, these results must be interpreted with caution, and the observed trends should be considered preliminary evidence requiring validation in larger-scale studies.

#### 5.1.2. Effect of Duodenal Infusion of L-Cit on Amino Acid Content in Ewes

L-lysine is an important amino acid whose excess in the feces indicates changes in the metabolism of microbes or deviations in the absorption in the host or a change in endogenous protein metabolism. L-lysine is absorbed in part in the small digestive tract and the rest passes through to the hindgut where it is subsequently passed on to the feces which leads to the degree of accumulation of fecal lysine [35]. Though L-Cit is not directly involved in the synthesis of L-lysine, it has an impact on the overall nitrogen metabolite and the microbiota in the feces and as a result indirectly affects the availability and metabolism of other amino acids. Improved efficacy of nitrogen usage conserves other amino acids to produce protein, whereas a change in fecal microflora can modify L-lysine’s degradation or synthesis of pathways. Indirectly, L-lysine is used to regulate the intestinal metabolism by promoting protein synthesis and nitrogen deposition [36]. It has been found that L-lysine supplementation significantly elevates the levels of serum total protein and albumin in lambs and reduces the level of urea nitrogen in the water, which is a strong indicator of better synthesis of nitrogen and a decline in the ammonia toxicity levels [37]. The results of the experiment quelled the findings of other studies that showed that fecal L-lysine increased significantly under duodenal L-Cit infusion, which supports the original suggestion that L-Cit may provoke amino acid production and metabolism in the body [15]. The significant increase in fecal L-lysine levels indicates that L-Cit can affect the intestinal microbial metabolism of particular amino acids or change the intestinal absorption capacity. The similarities between regions before and after the L-Cit intake of most urinary amino acids suggest that L-Cit does not cause increased nitrogen loss through the kidneys, consistent with the excess of nitrogen present in the body, probably reflected by whole proteins or peptides. Duodenal delivery of L-Cit could re-establish the metabolic pathways of nitrogen and amino acids in ewes, shifting the metabolic concentration away to anabolic metabolic pathways. This metabolic reprogramming is particularly important for ewes in reproductive cycles, i.e., during periods of pregnancy or lactation, because it aids in maintaining a positive nitrogen balance [38].

### 5.2. Effects of Duodenal Infusion of L-Cit on Plasma Metabolites in Ewes

Metabolomics technology provides a worldwide analysis of the metabolic situation of an organism, thus explaining small shifts caused by nutritional intercessions. Nucleotide and purine metabolism pathways play a significant role in controlling the metabolism of L-Cit in the intestines of sheep [39]. The enriched pathways indicated in this study were seven in total, with one of them being up-regulation in nucleotide and purine metabolism, and the down-regulation in linoleic acid metabolism. Nucleotides and purines are very important elements in the production of DNA, RNA and ATP and they are important in cell proliferation, energy metabolism and signal transduction [40]. The activities of the nucleotide metabolism pathway include the supply of substrates of nucleic acid synthesis [41], involvement in energy metabolism, control of signal transduction [42], and maintenance of cell proliferation, immune response, and redox balance [43]. The purine metabolism pathway is one of the key elements of nucleotide metabolism that mainly includes adenine and guanine. Synthesis of purines via the de novo pathway begins with PRPP and further becomes a series of enzyme reactions to form IMP, which is further metabolized to give AMP and GMP. The end product of this is uric acid, which results after the catalysis of hypoxanthine and xanthine by xanthine oxidase [44]. The purine metabolism pathway performs quite a number of essential functions, which include regulating the cellular energy levels via ATP and GTP [45], participating in repairing of the damage to DNA, as well as the antioxidant properties of the uric acid, which acts as a low-concentration antioxidant in the protection of blood vessels. These pathways may be up-regulated, which indicates that cellular energy status could be improved and processes that require rapid cell division, e.g., follicular growth and embryonic growth, could be promoted by duodenal infusion of L-Cit. The augmented production of nucleotides supplements essential forerunners of the fast increase in granulosa cells in folliculogenesis [46].

On the other hand, it was found that linoleic acid metabolism was down-regulated. Linoleic acid, being an ω-6 fatty acid, is a precursor to many eicosanoids that have important functions in inflammatory response and in reproductive processes [47]. The noted decrease in its metabolism suggests possible alterations in the lipid metabolism or inflammation process that can provide better conditions for reproductive processes. An overproduction of inflammatory reactions has been identified to affect the functioning of the ovary negatively and destroy embryo implantation [48]. A change in plasma metabolites indicates that the metabolic impacts of L-Cit are far-reaching and beyond its main role in the urea cycle, thus indicating the possibility of L-Cit to alter key metabolic processes linked with reproductive success. The intramuscular infusion of L-Cit in ewes in this study was noted to greatly improve the nucleotide and purine metabolism pathways. This improvement can be explained by a few processes: L-Cit can be used as a precursor of L-Arg and support the synthesis of NO through periodic cycling of arginine and dampening of the impact of the accumulation of ammonia through the urea cycle that affects the nucleotide metabolism indirectly [49]. Also, L-Cit enhances the supply of PRPP, which stimulates the synthesis of purine nucleotide production in the process of salvage and the deployment of related metabolic processes [42]. In addition, L-Cit improves the efficiency of mitochondrial metabolism by activating the enzymes of purine production via HIF-14 signaling pathways that lead to the build-up or faster breakdown of metabolic intermediates such as hypoxanthine [42]. Finally, with the help of the antioxidant effect of L-Cit, oxidative stress does not suppress the enzymes of nucleotide metabolism, thereby creating an active balance between the synthesis and the degradation of nucleotides [3]. The results in this experiment showed that the duodenal infusion of L-Cit resulted in a pronounced increase in the nucleotide metabolism pathway, linked to an increase in L-Arg metabolism.

### 5.3. Effects of Duodenal Infusion of L-Cit on Fecal Microbiota in Ewes

The fecal microflora is essential in the health and production performance of ruminants as it affects the digestion of nutrients, intake of energy, and metabolism in the host [50]. The paper has explored the effects of duodenal infusion of L-Cit on the fecal microbiome. Maslen [51] was proven that decreasing microbiome gene content and species abundance was strongly associated with an increase in feed efficiency and a lower alpha diversity, positively correlated with improved production performance. On the same note, Ma [52] reported that L-Cit addition reduced intestinal microbiota diversity in sheep by a significant margin and also had an excellent effect on production performance and species composition. It was found that L-Cit increased the level of hegemonic duodenal bacteria, thus promoting the intestine microbiota in the ewe. A notable change in microbial abundance generally altered the duodenal microbial community structure, indicating that L-Cit has positive influences on the duodesimal microbiota by regulating microbiota diversity. Moreover, energy acquisition and host metabolic condition are often correlated with changes in the *Firmicutes*-to-*Bacteroidetes* ratio [53]. An increasing number of *Firmicutes* indicates an increment in energy recovery of the feed.

In this work, the *Firmicutes*, *Bacteroidetes*, and *Proteobacteria* phyla were the most common in the intestine of the rumen after L-Cit delivery into the duodenum [54,55,56,57]. *Firmicutes* deal majorly with the degradation of fiber and cellulose [58]. *Bacteroidetes* are mainly engaged in the breakdown of complex carbohydrates and fermentation of organic matter [59]. Proteobacteria include pathogenic species like Escherichia coli, Salmonella, Vibrio cholerae and Helicobacter pylori, which cause diarrheal diseases in animals, among others [60]. Firmicutes as well as *Bacteroidetes* play a vital role in sugar and lipid metabolism, especially in the degradation of carbohydrates, which is vital to the overall health of the funnus and intestinal homeostasis. *Proteobacteria* are linked with diverse metabolic pathways and they can release various enzymes that deal with polysaccharide metabolism and protein metabolism, thus proving useful in the breakdown of polysaccharides and vitamins [61,62]. Li Xiaobin [63] noted that L-Cit when administered to mice by gavage led to a higher concentration of dominant phyla, including *Bacteroidetes*. This is an indication that L-Cit could possibly increase the proportion of good intestinal flora hence helping in maintaining the health of the intestines, which is similar to the results of the present research. Moreover, a declining tendency in the relative abundance of *Gastranaerophilales* was observed with the help of the differential discriminant analysis. This variation can be explained by the addition of L-Cit, which increases the total-body amino acid content, whereas *Gastranaerophilales* contributes to energy provision, signaling and host–microbiota interactions with the help of amino acid metabolism.

Correlation analysis showed a strong negative relationship between *Prevotellaceae* and its *UCG-004* branch and NO implying the participation of *Prevotellaceae* in the metabolism of L-Cit. This metabolism is a synergistic interaction between *Prevotellaceae* and *Gastranaerophilales*, which increase together to maintain host immune homeostasis [64]. Moreover, LEfSe revealed that there are strong differences in the abundances of *Planococcaceae*, *SP3-e08*, *Parvibacter*, *Anaerosporobacter*, *Butyricimonas*, and *Peptococcus*. This study indicates that L-Cit duodenal infusion shifts intestinal oxygen via the glycolysis/gluconeogenesis pathway and, in effect, it reduces its concentration and helps provide a favorable anaerobic climate to support the growth of favorable bacteria including *Parvibacter* and *Anaerosporobacter*. This condition can augment the growth and activation of intestinal immune cells [65], as well as maintain the balance and stability of the intestinal microecosystem by adjusting the metabolic activities of the microbes such as the generation of SCFAs [66]. *Akkermansiaceae* can degrade intestinal mucin to yield SCFAs present as acetate and propionate [67]. These fatty acids are important in maintaining intestinal immune homeostasis and an anaerobic environment, which is a requirement of obligate anaerobic symbionts and may promote a mutualistic relationship between the host and the symbiont [68]. The LEfSe indicated a number of taxa with a significant difference in their abundances. Though cyanobacteria are found in the intestine of mammals, they are not typically seen as being valuable, so their elimination is seen as a positive attribute. The percentage change in the *Planococcaceae* and *SP3-e08* indicates the growth of advantageous bacteria that participate in fermentation of carbohydrates and SCFA generation. These SCFAs are the energy sources for the host and can alter host metabolism and immune functioning [69].

The enrichment of butyrate-producing genera like *Butyricimonas* [70] may represent a potential mechanistic link, as butyrate is a short-chain fatty acid that can act as a histone deacetylase inhibitor and signaling molecule through G-protein coupled receptors, thereby potentially influencing host gene expression and neuroendocrine function.

### 5.4. Impact of Duodenal Infusion of L-Cit on Plasma Reproductive Hormone Levels in Ewes

Most of the direct actions of L-Cit on reproductive hormones are mediated via the NO metabolic pathway. In mammal cells, L-Cit gets converted successfully into L-Arg by the help of the enzymes AS and ASL [25]. GnRH is the first signal to the HPG axis initiating the secretion of FSH and LH by the anterior pituitary gland [71]. It is the rapid rise in GnRH, FSH, and LH observed in this study that formed first-hand evidence of the synergistic stimulation of the HPG axis by NO. LH is important in ovulation and corpus luteum development and maintenance, and FSH and LH via the blood vessels control ovaries to promote follicular growth, stimulate E_2_ production and release, and luteinize granulosa cells, thus maintaining P_4_ release by luteal cells [72]. Therefore, the fast activation of the upstream HPG axis through L-Cit via the NO pathway promotes ovulation and consequent corpus luteum development in ewes. It should be stated that GnRH commonly occurs in very low doses within the peripheral circulatory system and can be better quantified within hypothalamic–pituitary portal blood [73]. This is why the GnRH values used in the given study should be taken with a grain of salt. On the same note, FSH and LH are also secreted in a pulsatile fashion, and the sampling rates employed in the study might not have been good enough to determine actual peaks. Future research needs to conduct sampling more frequently to describe gonadotropin dynamics [74].

The significant height of P_4_ during 1,2 h after duodenal L-Cit infusion in the study is explained by the strong effect of heightened LH that directly stimulates the luteal cells and thus elevates the secretion of P_4_ [75]. Under luteal maintenance and P_4_ production, LH is instrumental. The low metabolic rate of L-Cit in the liver reduces the excess catabolism of L-Arg, thereby maintaining the production of the NO synthesis pathway, which indirectly contributes to the increase in P_4_. High levels of NO lead to an increase in P_4_ synthesis and release through participation in the improvement of blood circulation and luteal activities in the ovaries [76]. Moreover, the increase in P_4_ is highly accelerated, which could possibly be linked to a growth in adrenal-derived P_4_.

Compared with stimulation in the case of P_4_, GnRH, FSH, and LH, it is important to note that E_2_ levels decrease significantly after the infusion with duodenal L-Cit, which may serve as one of the indicators of a temporary inhibitory impact of aromatase caused by the upsurge in the level of L-Arg metabolites. Aromatase is a key enzyme in ovarian granulosa cells that converts the androgens to E_2_ [77,78]. In the case that metabolites of L-Cit inhibit the activity of aromatase, the production of E_2_ would then be affected. However, the neuroendocrine regulation of reproduction is complex and involves rapid feedback loops. E_2_ typically exerts a dual effect on GnRH secretion: low-to-moderate levels provide negative feedback, while sustained high levels during the late follicular phase trigger the positive feedback that leads to the preovulatory GnRH/LH surge [79]. In contrast, P_4_ predominantly exerts negative feedback on GnRH pulse frequency during the luteal phase, suppressing gonadotropin release [80]. Given this established paradigm, the concurrent increase in GnRH, FSH, and LH alongside a decrease in E_2_ observed in our study appears contradictory to the classical negative-feedback model, particularly the one primarily driven by P_4_. This divergence indicates that the reduced E_2_ levels are not the sole cause of, nor are they explained by, the expected negative feedback on GnRH. Moreover, a rise in the concentration of NO can increase hepatic blood flow, thus promoting the breakdown of E_2_. The liver has been reported to be the main location of E_2_ metabolism; E_2_ is readily cleared by the liver with the help of NO, which improves the chromic activity of hepatic blood circulation which leads to a low concentration of E_2_ in the blood. Furthermore, the involvement of antagonism, as well as synergy between E_2_ and E_2_, are also complex. Progesterone has the ability to cause its own receptors to be expressed, the activity of E_2_ receptors to be reduced by lowering its own concentration and at the same time inhibit the production of E_2_ [81]. The rapid increase in E_2_ level with the stimulation of L-Cit is indirectly linked to the decrease in the level of E_2_ by inhibiting either the production or activity of E_2_.

FSH and LH are secreted in a pulsatile fashion, and the sampling frequency employed in this study may not have been sufficient to capture their peak dynamics. Future studies should incorporate high-frequency (e.g., every 10–20 min) sampling to better characterize gonadotropin pulsatility. The results depict dynamic and time-varying changes in plasma reproductive hormone concentrations after the administration of L-Cit supplementation. In the current paper, the follicular rise in E_2_ after seven days of L-Cit supplementation indicated that E_2_ is critical in the formation of the follicles and subsequent estrous behavior. This rise in E_2_ is directly related to the rise in estrus rate that was being recorded. The follicles in development mainly synthesize estradiol which is important in estrous behavior and preparation of the uterus to be pregnant [82]. The inhibitory action of the high levels of E_2_ over the hypothalamus may be explained by the decrease in GnRH due to the negative-feedback mechanism. This first step suggests that L-Cit promotes early follicular development and E_2_ production, which triggers control over the reproductive system.

A major limitation of the present study is the lack of estrous cycle synchronization prior to the duodenal infusion experiment. This introduces a source of uncontrolled variability in baseline hormone concentrations. However, the random allocation of ewes to groups would be expected to distribute this variability evenly. Crucially, the acute and statistically significant surges in GnRH, FSH, LH, and P_4_ observed within 1–2 h of L-Cit infusion strongly indicate a treatment-specific effect superimposed upon any natural cyclical variation.

### 5.5. Effect of L-Cit Infusion into Duodenum on Correlation Analysis Between Fecal Microbiota and Amino Acid Content in Ewes

L-threonine is a crucial amino acid that is important in the synthesis of proteins and glycosylation of mucin, both of which are crucial to the health of the intestines [83]. The positive correlation that was observed between *Clostridia* and L-threonine means that such bacteria could be possibly involved in the production of L-threonine or increase the use of L-threonine by the host by means of improving the intestinal environment. On the other hand, the negative correlation at *Prevotellaceae* UCG-003 indicates that either of these bacterial groups may break down L-threonine or polypeptide absorption. As one of the most common non-essential amino acids in the body, L-glutamate is part of protein synthesis, the metabolism of energy, and the functioning of neurotransmitters [84]. Like L-threonine, *Clostridia* can either mediate the synthesis or use of L-glutamate in metabolic activity, yet *Prevotellaceae_UCG-003* appears to suppress these reactions. L-aspartic acid is an ingredient of the urea cycle and gluconeogenesis; it is a central component that is used in several metabolic processes [85]. The correlation trends identify the difference in host amino acid metabolism between *Clostridia* and *Prevotellaceae*. Moreover, L-lysine is a key protein-building amino acid and calcium absorption is also essential in collagen formation [86]. The increase in fecal L-lysine after infusion of L-Cit is related to the high frequency of *Clostridia* bacteria, which implies that the bacteria might promote L-lysine production of/or prevent its degradation by certain mechanisms. L-Arg, the immediate byproduct of L-Cit, is an essential precursor related to nitric oxide production [87]. This relationship suggests that communities of intestinal microbes and, especially, those containing *Clostridia*, can be involved in regulating L-Cit to L-Arg or the metabolism of L-Arg and, therefore, urinary L-Arg levels.

### 5.6. Effect of Duodenal Infusion of L-Cit on Correlation Analysis Between Fecal Microbiota and Plasma Metabolites in Ewes

The correlation analysis results indicated the effect of the fecal microbiota on the plasma metabolites. Hypoxanthine is a purine intermediate between the synthesis and degradation of nucleotides that had a positive association with *Clostridia* and a negative correlation with *Prevotellaceae* [40]. It implies that these microbial communities impact on host purine model metabolisms and, therefore, on the state of cells in terms of energy and growth potential, which play a vital role in reproductive processes like follicular development. Other purine metabolites, including inosine, which are linked with immunomodulatory and neuroprotective properties, were also linked to microbial communities [88]. This also gives more evidence to the hypothesis that L-Cit regulates host purine metabolism by means of microbial interactions.

The GnRH content exhibited a positive correlation of significant strength with the abundance of *Bacteroidaceae* and a negative correlation with the abundance of 8-Hydroxyquinoline and Cyclo-Dopa 5-O-Glucoside. The bacteria *Bacteroidaceae* is successful at breaking down complex polysaccharides and its products, including succinate, and is involved in the process of balancing GnRH through the gut–brain axis [89]. Moreover, the L-Cit that is transformed into arginine stimulates the mTOR pathway that increases hormone production and collaborates with metabolic processes of *Bacteroidaceae* [90]. As a chelating agent, 8-Hydroxyquinoline suppresses the activity of metal-dependent enzymes, which is why it suppresses the development of selected populations of bacteria. Its augmented expressions reduce the suppressive action of probiotics on GnRH secretion [91]. In addition, L-Cit affects the toxicity of 8-Hydroxyquinoline by means of altering the efficiency with which metal ions are utilized [92].

### 5.7. Effect of Duodenal Infusion of L-Cit on the Correlation Analysis Between Metabolites and Plasma Reproductive Hormones in Ewes

There was a significant positive correlation between the concentration of FSH and inosine levels and a negative correlation between the levels of the FSH and the abundance of the peptides Ile-Ile-Ile-Pro and Asn-Gln-Lys. An intermediate of the purine metabolism, inosine is able to increase the transduction of FSH by the A2A [93]. L-Cit is effective in the production of ATP through the arginine pathway, thus increasing the levels of inosine [94]. On the other hand, peptides like Ile-Ile-Ile-Pro and Asn-Gln-Lys inhibit the release of FSH because they switch the PARs in the intestine [95]. Also, L-Cit affects the rate of peptide hydrolysis by changing the intestinal PH that subsequently controls the amount of reproductive hormones [96]. Such metabolites help in the control of intestinal energy metabolism and barrier activity by the mTOR pathway.

The findings of the correlation test showed that plasma correlates regulate the plasma reproductive hormones. FSH is a gonadotropin released by the pituitary gland and its major activity is in follicular growth and development [97]. The fact that inosine and FSH have a positive correlation indicates that the activity of purine metabolism has an association with the formation and furthering of the follicles [98]. The proliferation of follicular cells needs to be as prompt as possible, and no one can do it without sufficient supply of nucleotides. The negative correlations of peptides Ile-Ile-Ile-Pro and Asn-Gln-Lys with FSH are found to be negative, which means that the peptides could prevent FSH secretion or action or could cause changes in body proteolysis in case of FSH elevation [73]. The most important controller of the idea of gonadotropin secretion by the pituitary gland is the GnRH secreted by the hypothalamus [99]. In addition, 8-hydroxyquinoline is also recognized as a chelating agent, which has antibacterial and anticancer effects. The negative relationship between Cyclo-Dopa 5-O-Glucoside and GnRH could indicate an inhibiting effect of the neuroendocrine system or it could be due to changes in certain metabolic processes when the levels of GnRH are high. In addition, such negative association indicates the possibility of Cyclo-Dopa 5-O-Glucoside being a player in GnRH negative feedback.

### 5.8. Effect of Duodenal Infusion of L-Cit on the Correlation Analysis Between Fecal Microbiota and Reproductive Hormones in Ewes

Correlation analysis has revealed that the phylum *Proteobacteria* covers many opportunistic pathogens and an augmented frequency is often associated with intestinal bleeding or ecological alteration [100]. The Greek phylum *Actinobacteriota* consists of bacteria with a variety of metabolic activities, among which are protein degradation, amino acid metabolic activities [101]. It was found that the phylum *Cyanobacteria* is correlated positively to it whereas the potentially harmful bacterial groups *Proteobacteria* and *Desulfobacteriaceae* are correlated negatively to it, which implies that the maintenance of intestinal health is supported by Actinobacteria. Despite the fact that *Pseudomonadota* is the general characteristic of a pathogen due to its opportunistic nature, it can also play a positive role in certain ecological niches [102]. Its strong associations with *Lachnospiraceae* and *Bacteroidetes* and adversarial associations with potentially pathogenic groups including *Desulfobacterota* and *Proteobacteria* highlight its complicated position in the intestinal ecosystem.

E_2_ is the most commonly produced estrogen by the ovarian granulosa cells and plays a role in follicular development and estrous behavior as well as preparing the uterus to begin pregnancy [82]. Research has shown that E_2_ can regulate host immune responses by stimulating release of anti-inflammatory cytokines, inhibiting Proteobacteria growth and preserving intestinal barrier integrity [103]. Moreover, L-Cit affects intestinal redox status via the pathway of NO where it suppresses the growth of *Proteobacteria* indirectly. Some species of Cyanobacteria have nitrogen-fixing abilities, which also contributes to nitrogen sources that promote the production of arginine through L-Cit metabolism stimulating the intestinal urea cycle and supplementing the E_2_ metabolic regulation of the process [104]. *Oscillospiraceae* are the major producers of butyrate and their abundance normally has a positive relationship with intestinal health [105]. Nevertheless, E_2,_ by regulating host energy metabolism, decreases the butyrate need leading to lower abundance of *Oscillospiraceae* [106]. In addition, L-Cit indirectly regulates the microbial composition of the intestines by affecting SCFA-use efficiency [107]. Intestinal microorganisms have the ability to metabolize estrogen and thus androgenically affect enterohepatic circulation and systemic concentrations [108]. The negative correlations emphasize the complexity of the regulatory network of microbial communities on host female reproductive hormones.

GnRH, a key hormone released by the hypothalamus, is very vital in controlling the release of pituitary gonadotropins, which are FSH and LH, hence it also controls ovarian functioning [73]. Bacteroides, a common genus in fecal content, is a compartment of carbohydrate breakdown as well as the creation of a variety of metabolites [109]. A positive relationship between GnRH and Bacteroides is observed suggesting that the metabolic activities of Bacteroides can positively influence the production or release of GnRH by some means, and thus the HPG axis. The interaction may well include neuropathogenic substances generated through the work of microorganisms or indirect action on the hypothalamus through links with the immune system [110].

It is critical to note that these statistical associations do not constitute evidence for a causal, mechanistic link. Targeted studies using germ-free models, specific microbial inoculations, or pathway inhibitors would be required to validate the regulatory roles of these specific microbiota–metabolite–hormone axes suggested by our correlative findings.

**Figure 11:**
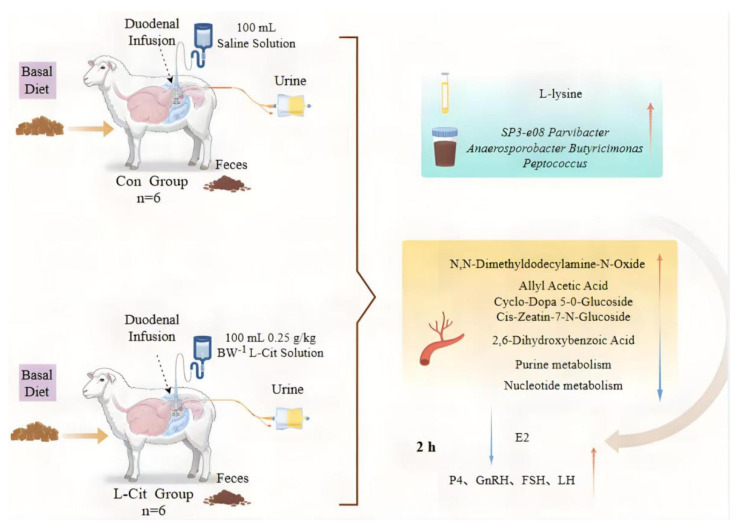
Effects of duodenal infusion of L-Cit on plasma metabolism, fecal microbiota structure, and reproductive hormones in ewes.

These metabolic shifts were linked to the enhanced secretion of key reproductive hormones, including GnRH, FSH, LH, and P_4_, suggesting a mechanistic basis for supporting reproductive endocrine function in ewes. (Figure 11).

## 6. Conclusions

Under the experimental conditions of this study, supplementation with L-Cit altered the gastrointestinal microbiota, significantly increasing the abundance of beneficial bacteria such as intestinal genera including *SP3-e08*, *Parvibacter*, and *Butyricimonas*. These microbial changes effectively enhanced nitrogen metabolism and amino acid utilization efficiency in ewes, markedly reducing fecal and urinary nitrogen excretion and improving nitrogen retention. In blood metabolism, L-Cit supplementation significantly up-regulated the purine metabolism and nucleotide metabolism pathways, thereby enhancing cellular energy supply, nucleic acid synthesis, and proliferative capacity. Concurrently, the linoleic acid metabolism pathway was down-regulated. These metabolic shifts collectively promoted the secretion of key reproductive hormones, including GnRH, FSH, LH, and P_4_, ultimately leading to improved reproductive performance in the ewes.

## Figures and Tables

**Table 1:** Composition and nutritional levels of the basal diet (dry matter basis)%.

Ingredients	Content	Nutrient Levels	Content
Whole corn silage	35.15	DM	59.26
Corn husk	35.15	CP	12.87
Premix ^(1)^	15.07	EE	3.08
Sorghum stalks	7.54	Ash	5.54
30-peptide	6.21	NDF	26.54
NaHCO_3_	0.51	ADF	14.03
NaCl	0.31	Ca	0.43
Vitamin D3	0.03	P	0.36
Sodium selenite	0.03	ME (MJ/kg) ^(2)^	10.85

Note: ^(1)^ The premix provided the following per kg of the concentrate supplement. Fe (as ferrous sulfate) 20.5 mg, Zn (as zinc sulfate) 23.2 mg, Cu (as copper sulfate) 5.7 mg, Se (as sodium selenite) 0.7 mg, Ca (as calcium iodate) 1.3 mg, Mg (as magnesium oxide) 0.4 mg, Co (as cobalt chloride) 3.6 mg, VA 6000.0 IU, VD 30.7 mg, VE 8.0 mg. ^(^^2)^ Metabolic energy is calculated, other nutrient levels were measured values.

**Table 2:** Feed intake, water intake, total L-Cit infusion, total fecal output, and total urine output.

Item	Con Group	*L*-Cit Group	*p*-Value
Feed intake (kg/d)	1.48 ± 0.51	1.56 ± 0.37	0.719
Water intake (L/d)	1.53 ± 0.74	1.69 ± 0.63	0.645
Total *L*-Cit infusion volume (g/d)	0	7.86 ± 1.14	
Total fecal matter (kg/d)	0.93 ± 0.30	0.73 ± 0.31	0.282
Total volume of urine (L/d)	0.53 ± 0.17	0.42 ± 0.12	0.267

**Table 3:** Effects of duodenal L-Cit infusion on crude protein in ewes.

Item	Con Group	*L*-Cit Group	*p*-Value
Feces (DM)	96.98 ± 0.00269%	96.97 ± 0.00049%	>0.05
Feces (CP)	10.62 ± 0.389% ^a^	9.35 ± 0.134% ^b^	<0.05
Urine (CP)	6.17 ± 0.199% ^A^	4.06 ± 0.193% ^B^	<0.01

Note: $\omega \;=\;\frac{(V_{2}\;-{\;V}_{1})\;\times \;c\;\times \;\frac{14}{1000\;}\times \;6.25}{m\;\times \;\frac{V^{\prime }}{V}}\;\times \;100$%.

**Table 4:** Comparison of nitrogen metabolism indicators in ewes after duodenal L-Cit infusion.

Item	Con Group	*L*-Cit Group	*p*-Value
Nitrogen uptake (g/d)	24.35	24.35	1
Fecal nitrogen (g/d)	13.91	12.36	0.6
Urine nitrogen (g/d)	4.21	3.32	0.266
*L*-Cit nitrogen content (g/d)	0	1.89	
Total nitrogen discharge (g/d)	18.12	15.77	0.464
Sedimentary nitrogen (g/d)	9.08	12.39	0.227
Nutrient utilization efficiency %	32.50	48.80	0.096
Nitrogen biological value %	64.69	77.58	0.107

Note: Nitrogen intake (g/d) = DMI × dietary DM nitrogen content; fecal nitrogen (g/d) = (fecal CP content × daily fecal output)/6.25; urinary nitrogen (g/d) = (urinary CP content × daily urine output)/6.25; total nitrogen excretion (g/d) = fecal nitrogen + urinary nitrogen; retained nitrogen (g/d) = nitrogen intake − fecal nitrogen − urinary nitrogen; nitrogen utilization rate (%) = 100% × retained nitrogen/nitrogen intake; biological value of nitrogen (%) = 100% × retained nitrogen/(nitrogen intake − fecal nitrogen).

## Data Availability

The datasets presented in this study can be found in the NCBI repository under BioProject accession number PRJNA1290497, available at http://www.ncbi.nlm.nih.gov/bioproject/PRJNA1290497 (accessed on 20 March 2026).

## Abbreviations

ADF	Acid Detergent Fiber
BW	Body Weight
CP	Crude Protein
DM	Dry Matter
E_2_	Estradiol
EE	Ether Extract
FSH	Follicle-Stimulating Hormone
GnRH	Gonadotropin-Releasing Hormone
L-Cit	L-Citrulline
LH	Luteinizing Hormone
NO	Nitric Oxide
P_4_	Progesterone
RNA	Ribonucleic Acid
TMR	Total Mixed Ration

## References

[1] Papadia C., Osowska S., Cynober L. (2018). Citrulline in health and disease. Review on human studies. Clin. Nutr..

[2] Summar M. (2024). Potential therapeutic uses of L-citrulline beyond genetic urea cycle disorders. J. Inherit. Metab. Dis..

[3] Aguayo E., Martínez-Sánchez A., Fernández-Lobato B., Alacid F. (2021). L-Citrulline: A Non-Essential Amino Acid with Important Roles in Human Health. Appl. Sci..

[4] Janjić M.M., Stojkov N.J., Andrić S.A., Kostic T.S. (2012). Anabolic-androgenic steroids induce apoptosis and NOS2 (nitric-oxide synthase 2) in adult rat Leydig cells following in vivo exposure. Reprod. Toxicol..

[5] Wagemaker M.J., Eastwood D.C., van der Drift C., Jetten M.S., Burton K., Van Griensven L.J., Op den Camp H.J. (2007). Argininosuccinate synthetase and argininosuccinate lyase: Two ornithine cycle enzymes from Agaricus bisporus. Mycol. Res..

[6] Juan C.A., Pérez de la Lastra J.M., Plou F.J., Pérez-Lebeña E. (2021). The Chemistry of Reactive Oxygen Species (ROS) Revisited: Outlining Their Role in Biological Macromolecules (DNA, Lipids and Proteins) and Induced Pathologies. Int. J. Mol. Sci..

[7] Wu G., Meininger C.J., McNeal C.J., Bazer F.W., Rhoads J.M. (2021). Role of L-Arginine in Nitric Oxide Synthesis and Health in Humans. Adv. Exp. Med. Biol..

[8] Cormio L., De Siati M., Lorusso F., Selvaggio O., Mirabella L., Sanguedolce F., Carrieri G. (2011). Oral L-citrulline supplementation improves erection hardness in men with mild erectile dysfunction. Urology.

[9] Visek W.J. (1984). An update of Concepts of essential amino acids. Annu. Rev. Nutr..

[10] Zhao G., Zhao X., Bai J., Dilixiati A., Song Y., Haire A., Zhao S., Aihemaiti A., Fu X., Wusiman A. (2023). Metabolomic and Transcriptomic Changes Underlying the Effects of L-Citrulline Supplementation on Ram Semen Quality. Animals.

[11] Fan C., Aihemaiti A., Fan A., Dilixiati A., Zhao X., Li Z., Chen C., Zhao G. (2024). Study on the correlation of supplementation with L-citrulline on the gastrointestinal flora and semen antifreeze performance of ram. Front. Microbiol..

[12] Ma Y., Zhao G., Wang C. (2023). Effects of supplementation with different Concentrations of L-Citrulline on the plasma amino acid Concentration, reproductive hormone Concentrations, antioxidant capaCity, and reproductive performance of Hu ewes. Anim. Prod. Sci..

[13] Gilbreath K.R., Nawaratna G.I., Wickersham T.A., Carey S.M., Bazer F.W., Wu G. (2021). Ruminal microbes of adult steers do not degrade extracellular L-citrulline and have a limited ability to metabolize extracellular L-glutamate. J. Anim. Sci..

[14] Gilbreath K.R., Bazer F.W., Carey S.M., Cleere J.J., Wu G. (2020). Ruminal microbes of adult sheep do not degrade extracellular L-citrulline. J. Anim. Sci..

[15] Zhao D., Gao Y., Chen Y., Zhang Y., Deng Y., Niu S., Dai H. (2024). L-Citrulline Ameliorates Iron Metabolism and Mitochondrial Quality Control via Activating AMPK Pathway in Intestine and Improves Microbiota in Mice with Iron Overload. Mol. Nutr. Food Res..

[16] An M., Ma Y., Liu Z. (2022). Effects of supplementation with arginine on reproductive and lactation performance in 90-day pregnant Hu sheep. China Feed.

[17] Lynch G.L., Klusmeyer T.H., Cameron M.R., Clark J.H., Nelson D.R. (1992). Effects of somatotropin and duodenal infusion of amino acids on nutrient passage to duodenum and performance of dairy cows. J. Dairy Sci..

[18] Klepacki J., Klawitter J., Klawitter J., Karimpour-Fard A., Thurman J., Ingle G., Patel D., Christians U. (2016). Amino acids in a targeted versus a non-targeted metabolomics LC-MS/MS assay. Are the results Consistent?. Clin. Biochem..

[19] Di Segni A., Braun T., BenShoshan M., Farage Barhom S., Glick Saar E., Cesarkas K., Squires J.E., Keller N., Haberman Y. (2018). Guided Protocol for Fecal Microbial Characterization by 16S rRNA-AmpliCon Sequencing. J. Vis. Exp..

[20] Zeng Y., Luo L., Hou W. (2017). Targeted metabolomics analysis of aromatic amino acids and their gut microbiota–host cometabolites in rat serum and urine by liquid chromatography coupled with tandem mass spectrometry. J. Sep. Sci..

[21] Morizono H., Caldovic L., Shi D., Tuchman M. (2004). Mammalian N-acetylglutamate synthase. Mol. Genet. Metab..

[22] Wu G., Davis P.K., Flynn N.E. (1997). Endogenous synthesis of arginine plays an important role in maintaining arginine homeostasis in post weaning growing pigs. J. Nutr..

[23] Tujioka K., Lyou S., Hirano E., Sano A., Hayase K., Yoshida A., Yokogoshi H. (2002). Role of N-acetylglutamate Concentration and ornithine transport into mitochondria in urea synthesis of rats given proteins of different quality. J. Agric. Food Chem..

[24] Yu Y.G., Turner G.E., Weiss R.L. (1996). Acetylglutamate synthase from Neurospora crassa: Structure and regulation of expression. Mol. Microbiol..

[25] Morris S.M. (2002). Regulation of enzymes of the urea cycle and arginine metabolism. Annu. Rev. Nutr..

[26] Levillain O., Hus-Citharel A., Morel F. (1990). Localization of arginine synthesis along rat nephron. Am. J. Physiol..

[27] Husson A., Brasse-Lagnel C., Fairand A. (2003). Argininosuccinate synthetase from the urea cycle to the citrulline-NO cycle. Eur. J. Biochem..

[28] Fu B., Danzeng B., Zhao X. (2024). Effects of different feed metabolic energy and protein levels on growth performance, energy and nitrogen metabolism, and serum biochemical indicators in Yunshang black goats during the growth period. J. Anim. Nutr..

[29] Cynober L., de Bandt J.P., Moinard C. (2010). The 2009 ESPEN Sir David Cuthbertson. Citrulline: A new major signaling molecule or just another player in the pharmaconutrition game?. Clin. Nutr..

[30] Li Y. (2021). The role of purinergic signaling in microglial responses. Stress Brain.

[31] Chojnacka K., Mikula K., Izydorczyk G., Skrzypczak D., Witek-Krowiak A., Gersz A., Moustakas K., Iwaniuk J., Grzędzicki M., Korczyński M. (2021). Innovative high digestibility protein feed materials reducing environmental impact through improved nitrogen-use efficiency in sustainable agriculture. J. Environ. Manag..

[32] Wu G. (2013). Amino acids: Metabolism, functions, and nutrition. Amino Acids.

[33] Du J., Gan M., Xie Z., Zhou C., Jing Y., Li M., Liu C., Wang M., Dai H., Huang Z. (2023). Effects of dietary L-Citrulline supplementation on growth performance, meat quality, and fecal microbial composition in finishing pigs. Front. Microbiol..

[34] Lacroix M., Ouellet D.R., Lapierre H. (2010). Effects of dietary arginine on nitrogen metabolism in lactating dairy cows. J. Dairy Sci..

[35] Luo J., Chen D. (2006). Effects of Lysine on Protein Metabolism and Its Potential Regulatory Mechanisms. Feed Ind..

[36] Blachier F., Boutry C., Sansonetti P.J., van Hylckama Vlieg J.E.T. (2019). The gut microbiota and amino acid metabolism. Curr. Opin. Clin. Nutr. Metab. Care.

[37] Cheng S., Li J., Feng R. (2010). Effects of Lysine Coating on Nitrogen Digestion and Metabolism in Sheep. Anhui Agric. Sci..

[38] Biggs C.R., Yeager L.A., Bolser D.G., Bonsell C., Dichiera A.M., Hou Z., Keyser S.R., Khursigara A.J., Lu K., Muth A.F. (2020). Does functional redundancy affect ecological stability and resilience?. A Rev. Meta-Anal..

[39] Moffatt B.A., Ashihara H. (2002). Purine and pyrimidine nucleotide synthesis and metabolism. Arab. Book.

[40] Lane N., Fan T.W.M. (2015). Regulation of mammalian nucleotide metabolism and biosynthesis. Nucleic Acids Res..

[41] Zhu Y., Tong X., Xue J., Qiu H., Zhang D., Zheng D.Q., Tu Z.C., Ye C. (2025). Phospholipid biosynthesis modulates nucleotide metabolism and reductive capacity. Nat. Chem. Biol..

[42] Ali E.S., Ben-Sahra I. (2023). Regulation of nucleotide metabolism in cancers and immune disorders. Trends Cell Biol..

[43] Shi D.D., Savani M.R., Abdullah K.G., McBrayer S.K. (2023). Emerging roles of nucleotide metabolism in cancer. Trends Cancer.

[44] National Center for Biotechnology Information PubChem Pathway Summary for Pathway SMP0000050, Purine Metabolism, Source: PathBank. https://pubchem.ncbi.nlm.nih.gov/pathway/PathBank:SMP0000050.

[45] Pedley A.M., Benkovic S.J. (2017). A New View into the Regulation of Purine Metabolism: The Purinosome. Trends Biochem. Sci..

[46] Tang Z., Ye W., Chen H., Kuang X., Guo J., Xiang M., Peng C., Chen X., Liu H. (2019). Role of purines in regulation of metabolic reprogramming. Purinergic Signal..

[47] Funk C.D. (2001). Prostaglandins and leukotrienes: Advances in eicosanoid biology. Science.

[48] Brännström M., Norman R.J. (1993). Involvement of leukocytes and cytokines in the ovulatory process and corpus luteum function. Hum. Reprod..

[49] Sureda A., Pons A. (2012). Arginine and citrulline supplementation in sports and exercise: Ergogenic nutrients?. Med. Sci. Sports Exerc..

[50] Jami E., White B.A., Mizrahi I. (2013). The rumen microbiome as a source of innovation for improving the efficiency of feed utilization in ruminants. Environ. Microbiol..

[51] Maslen B.N., Duff C., Clark S.A., Van der Werf J., White J.D., Pant S.D. (2023). Increased yearling weight gain is associated with a distinct Faecal microbial profile. Animals.

[52] Ma Y., Liu T., Chen S., Shen H., Wang J. (2025). Dietary supplementation with L-citrulline improves amino acid composition and broiler performance, and modulates gut microbiota. Front. Microbiol..

[53] Turnbaugh P.J., Ley R.E., Mahowald M.A., Magrini V., Mardis E.R., Gordon J.I. (2006). An obesity-associated gut microbiome with increased capacity for energy harvest. Nature.

[54] Ley R.E., Lozupone C.A., Hamady M., Knight R., Gordon J.I. (2008). Worlds within worlds: Evolution of the vertebrate gut microbiota. Nat. Rev. Microbiol..

[55] Callaway T.R., Dowd S.E., Edrington T.S., Anderson R.C., Krueger N., Bauer N., Kononoff P.J., Nisbet D.J. (2010). Evaluation of bacterial diversity in the rumen and feces of cattle fed different levels of dried distillers grains plus solubles using bacterial tag-encoded FLX ampliCon pyrosequencing. J. Anim. Sci..

[56] de Menezes A.B., Lewis E., O’Donovan M., O’Neill B.F., Clipson N., Doyle E.M. (2011). Microbiome analysis of dairy cows fed pasture or total mixed ration diets. FEMS Microbiol. Ecol..

[57] Mao S., Zhang M., Liu J., Zhu W. (2015). Characterising the bacterial microbiota across the gastrointestinal tracts of dairy cattle: Membership and potential function. Sci. Rep..

[58] Langille M.G., Zaneveld J., Caporaso J.G., McDonald D., Knights D., Reyes J.A., Clemente J.C., Burkepile D.E., Vega Thurber R.L., Knight R. (2013). Predictive functional profiling of microbial communities using 16S rRNA marker gene sequences. Nat. Biotechnol..

[59] Reigstad C.S., Kashyap P.C. (2013). Beyond phylotyping: Understanding the impact of gut microbiota on host biology. Neurogastroenterol. Motil..

[60] Li X., Yang L., Huo G. (2012). Research on differences in gut microbiota of infants with different feeding methods using Illumin technology. Food Sci. Technol..

[61] Abdul Rahman N., Parks D.H., Vanwonterghem I., Morrison M., Tyson G.W., Hugenholtz P. (2016). A phylogenomic analysis of the bacterial phylum Fibrobacteres. Front. Microbiol..

[62] Colston T.J., Jackson C.R. (2016). Microbiome evolution along divergent branches of the vertebrate tree of life: What is known and unknown. Mol. Ecol..

[63] Li X.B., Zhao G.D., Qiu H.R., Abudukahaer A., Aizezi M., Ai L. (2019). Effects of L-Citrulline on Body Weight and Intestinal Flora Diversity of Mice. Chin. J. Anim. Nutr..

[64] Martinez-Guryn K., Leone V., Chang E.B. (2019). Regional Diversity of the Gastrointestinal Microbiome. Cell Host Microbe.

[65] Ilinskaya O.N., Ulyanova V.V., Yarullina D.R., Gataullin I.G. (2017). Secretome of intestinal Bacilli: A natural guard against pathologies. Front. Microbiol..

[66] Yang L., Zhao Y., Huang J., Zhang H., Lin Q., Han L. (2020). Insoluble dietary fiber from soy hulls regulates the gut microbiota in vitro and increases the abundance of bifidobacteriales and lactobacillales. J. Food Sci. Technol..

[67] Feng W., Ao H., Peng C. (2018). Gut microbiota, short-chain fatty acids, and herbal medicines. Front. Pharmacol..

[68] Shealy N.G., Yoo W., Byndloss M.X. (2021). Colonization resistance: Metabolic warfare as a strategy against pathogenic Enterobacteriaceae. Curr. Opin. Microbiol..

[69] Morrison D.J., Preston T. (2016). Formation of short chain fatty acids by the gut microbiota and their impact on human metabolism. Gut Microbes..

[70] Hotta Y., Shiota A., Kataoka T., Motonari M., Maeda Y., Morita M., Kimura K. (2014). Oral L-citrulline supplementation improves erectile function and penile structure in castrated rats. Int. J. Urol..

[71] Ashley R.L., Arreguin-Arevalo J.A., Nett T.M. (2009). Binding characteristics of the ovine membrane progesterone receptor alpha and expression of the receptor during the estrous cycle. Reprod. Biol. Endocrinol..

[72] Clarke I.J., Cummins J.T. (1982). The temporal relationship between GnRH and LH secretion in ewes. Endocrinology.

[73] Goodman R.L., Gibson M., Skinner D.C., Lehman M.N. (2022). Neuroendocrine control of pulsatile GnRH secretion during the ovarian cycle: Evidence from the ewe. Reprod Suppl..

[74] Niswender G.D., Juengel J.L., Silva P.J., Rollyson M.K., McIntush B.C. (2000). Mechanisms Controlling the function and life span of the corpus luteum. Physiol. Rev..

[75] Sigaud S., Goldsmith C.A.W., Zhou H., Yang Z., Fedulov A., Lmrich A., Kobzik L. (2007). Air pollution particles diminish bacterial clearance in the primed lungs of mice. Toxicol. Appl. Pharmacol..

[76] Dart D.A., Waxman J., Aboagye E.O., Bevan C.L. (2013). Visualising androgen receptor activity in male and female mice. PLoS ONE.

[77] Davis S.R., Tran J. (2001). Testosterone influences libido and well being in women. Trends Endocrinol. Metab..

[78] Karsch F.J. (1987). Steroid modulation of GnRH secretion. Recent Prog. Horm. Res..

[79] Williams G.L., Cardoso R.C. (2021). Neuroendocrine control of estrus and ovulation. Bovine Reproduction.

[80] Rekawiecki R., Kowalik M.K., Kotwica J. (2017). The expression of progesterone receptor coregulators mRNA and protein in corpus luteum and endometrium of cows during the estrous cycle. Anim. Reprod. Sci..

[81] Richards J.S., Russell D.L., Robker R.L., Dajee M., Filipovits M. (1998). Molecular mechanisms of ovulation and luteinization. Mol. Cell. Endocrinol..

[82] Wu G., Bazer F.W., Satterfield M.C., Li X., Wang X., Johnson G.A., Burghardt R.C., Dai Z., Wang J., Wu Z. (2013). Impacts of arginine nutrition on embryonic and fetal development in mammals. Amino Acids.

[83] Newsholme P., Leite V. (2010). Glutamine and glucose as fuels for immune cells. Braz. J. Med. Biol. Res..

[84] Brosnan J.T., Brosnan M.E. (2006). The catabolism of amino acids and the urea cycle. J. Nutr..

[85] Reeds P.J. (2000). Dispensable and indispensable amino acids for humans. J. Nutr..

[86] Wu G., Bazer F.W., Davis T.A., Kim S.W., Li P., Marc Rhoads J., Spencer T.E. (2009). Arginine metabolism and nutrition in growth, health and disease. Amino Acids.

[87] Haskó G., Cronstein B. (2013). Regulation of Inflammation by Adenosine. Front. Immunol..

[88] McKee L.S., La Rosa S.L., Westereng B., Eijsink V.G., Pope P.B., Larsbrink J. (2021). Polysaccharide degradation by the Bacteroidetes: Mechanisms and nomenclature. Environ. Microbiol. Rep..

[89] Geiger R., Rieckmann J.C., Wolf T., Basso C., Feng Y., Fuhrer T., Kogadeeva M., Picotti P., Meissner F., Mann M. (2016). L-Arginine Modulates T Cell Metabolism and Enhances Survival and Anti-tumor Activity. Cell.

[90] Prachayasittikul V., Prachayasittikul S., Ruchirawat S., Prachayasittikul V. (2013). 8-Hydroxyquinolines: A review of their metal chelating properties and medicinal applications. Drug Des. Dev. Ther..

[91] Pape V.F.S., Gaál A., Szatmári I., Kucsma N., Szoboszlai N., Streli C., Fülöp F., Enyedy É.A., Szakács G. (2021). Relation of Metal-Binding Property and Selective Toxicity of 8-Hydroxyquinoline Derived Mannich Bases Targeting Multidrug Resistant Cancer Cells. Cancers.

[92] Welihinda A.A., Kaur M., Greene K., Zhai Y., Amento E.P. (2016). The adenosine metabolite inosine is a functional agonist of the adenosine A2A receptor with a unique signaling bias. Cell. Signal..

[93] Goron A., Lamarche F., Blanchet S., Delangle P., Schlattner U., Fontaine E., Moinard C. (2019). Citrulline stimulates muscle protein synthesis, by reallocating ATP Consumption to muscle protein synthesis. J. Cachexia Sarcopenia Muscle.

[94] Nie C., He T., Zhang W., Zhang G., Ma X. (2018). Branched Chain Amino Acids: Beyond Nutrition Metabolism. Int. J. Mol. Sci..

[95] Nasri M. (2017). Protein Hydrolysates and Biopeptides: Production, Biological Activities, and Applications in Foods and Health Benefits. Adv. Food Nutr. Res..

[96] Findlay J.K. (1993). An update on the roles of inhibin, activin, and follistatin as local regulators of ovarian function. Biol. Reprod..

[97] Downs S.M., Hudson E.D. (2001). Purine metabolism and oocyte maturation. Mol. Reprod. Dev..

[98] Ghasemi S., Ghasemi J.B. (2017). 8-Hydroxyquinoline: A versatile ligand in coordination chemistry and its applications. Coord. Chem. Rev..

[99] Bao R., Hesser L.A., He Z., Zhou X., Nadeau K.C., Nagler C.R. (2021). Fecal microbiome and metabolome differ in healthy and food-allergic twins. J. Clin. Investig..

[100] Ventura M., Canchaya C., Fitzgerald G.F., Zink R., van Sinderen D. (2007). Genomics of Actinobacteria: Insights into milk fermentation and probiotic properties. Antonie Van Leeuwenhoek.

[101] Klockgether J., Tümmler B. (2017). Pseudomonas aeruginosa in cystic fibrosis: Pathogenesis and treatment. FEMS Microbiol. Rev..

[102] Rosario D., Bidkhori G., Lee S. (2021). Systematic analysis of gut microbiome reveals the role of bacterial folate and homocysteine metabolism in Parkinson’s disease. Cell Rep..

[103] Guillaume M.C., Branco Dos Santos F. (2023). Assessing and reducing phenotypic instability in cyanobacteria. Curr. Opin. Biotechnol..

[104] Konikoff T., Gophna U. (2016). Oscillospira: A Central, Enigmatic Component of the Human Gut Microbiota. Trends Microbiol..

[105] Feng W., Liu J., Ao H., Yue S., Peng C. (2020). Targeting gut microbiota for precision medicine: Focusing on the efficacy and toxicity of drugs. Theranostics.

[106] Ma J., Piao X., Mahfuz S., Long S., Wang J. (2021). The interaction among gut microbes, the intestinal barrier and short chain fatty acids. Anim. Nutr..

[107] Plottel C.S., Blaser M.J. (2011). Microbiome and malignancy. Cell Host Microbe.

[108] Wexler H.M. (2007). Bacteroides: The good, the bad, and the nitty-gritty. Clin. Microbiol. Rev..

[109] Cryan J.F., Dinan T.G. (2012). Mind-altering microorganisms: The impact of the gut microbiota on brain and behaviour. Nat. Rev. Neurosci..

[110] Dalile B., Van Oudenhove L., Vervliet B., Verbeke K. (2019). The role of short-chain fatty acids in microbiota–gut–brain communication. Nat. Rev. Gastroenterol. Hepatol..

